# Directed Gas-Phase Formation of Azulene (C_10_H_8_): Unraveling the Bottom-Up Chemistry of Saddle-Shaped Aromatics

**DOI:** 10.1021/acscentsci.4c01606

**Published:** 2025-02-04

**Authors:** Zhenghai Yang, Kazuumi Fujioka, Galiya R. Galimova, Iakov A. Medvedkov, Shane J. Goettl, Rui Sun, Alexander M. Mebel, Ralf I. Kaiser

**Affiliations:** † Department of Chemistry, University of Hawai’i at Manoa, Honolulu, Hawaii 96822, United States; ‡ Department of Chemistry and Biochemistry, Florida International University, Miami, Florida 33199, United States

## Abstract

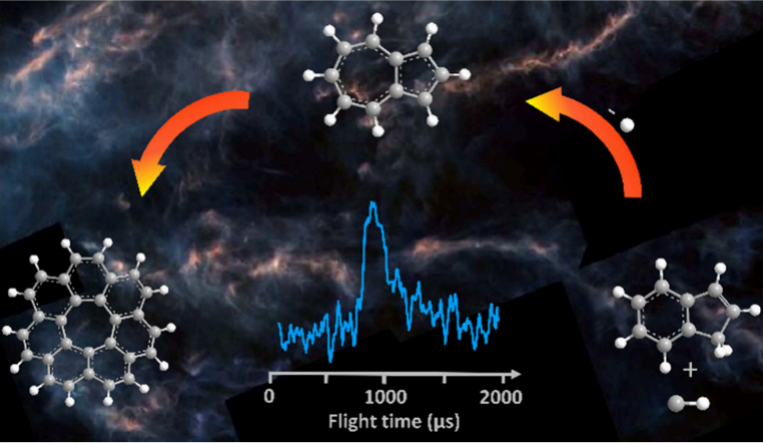

The azulene (C_10_H_8_) molecule, the simplest polycyclic aromatic hydrocarbon (PAH) carrying a fused seven- and five-membered ring, is regarded as a fundamental molecular building block of saddle-shaped carbonaceous nanostructures such as curved nanographenes in the interstellar medium. However, an understanding of the underlying gas-phase formation mechanisms of this nonbenzenoid 10π-Hückel aromatic molecule under low-temperature conditions is in its infancy. Here, by merging crossed molecular beam experiments with electronic structure calculations and molecular dynamics simulations, our investigations unravel an unconventional low-temperature, barrierless route to azulene via the reaction of the simplest organic radical, methylidyne (CH), with indene (C_9_H_8_) through ring expansion. This reaction might represent the initial step toward to the formation of saddle-shaped PAHs with seven-membered ring moieties in hydrocarbon-rich cold molecular clouds such as the Taurus Molecular Cloud-1 (TMC-1). These findings challenge conventional wisdom that molecular mass growth processes to nonplanar PAHs, especially those containing seven-membered rings, operate only at elevated pressure and high-temperature conditions, thus affording a versatile low-temperature route to contorted aromatics in our galaxy.

## Abstract 2

The azulene (C_10_H_8_) molecule, a fundamental molecular building block of saddle-shaped aromatics, is formed for the first time under single collision conditions.

## Introduction

Since the synthesis of the very first nonbenzenoid, 10π-Hückel aromatic azulene molecule (C_10_H_8_) carrying a seven-membered ring fused with a five-membered cyclopentadienyl moiety from Δ^9^-actalin in 1937,^1^ aromatic molecules with five-, six-, and seven-membered rings have garnered fundamental interest in the realm of combustion chemistry and astrochemistry as molecular building blocks to 2-dimensional,^2,3^ 3-dimensional,^4^ and saddle-shaped^5^ carbonaceous nanomaterials eventually leading to soot particles (combustion)^6−8^ and interstellar grains (astrochemistry).^9−11^ However, the distinct mechanistic frameworks of the molecular mass growth processes involving five-, six-, and seven-membered ring moieties represent a fundamental unsolved puzzle.

**Figure 1:**
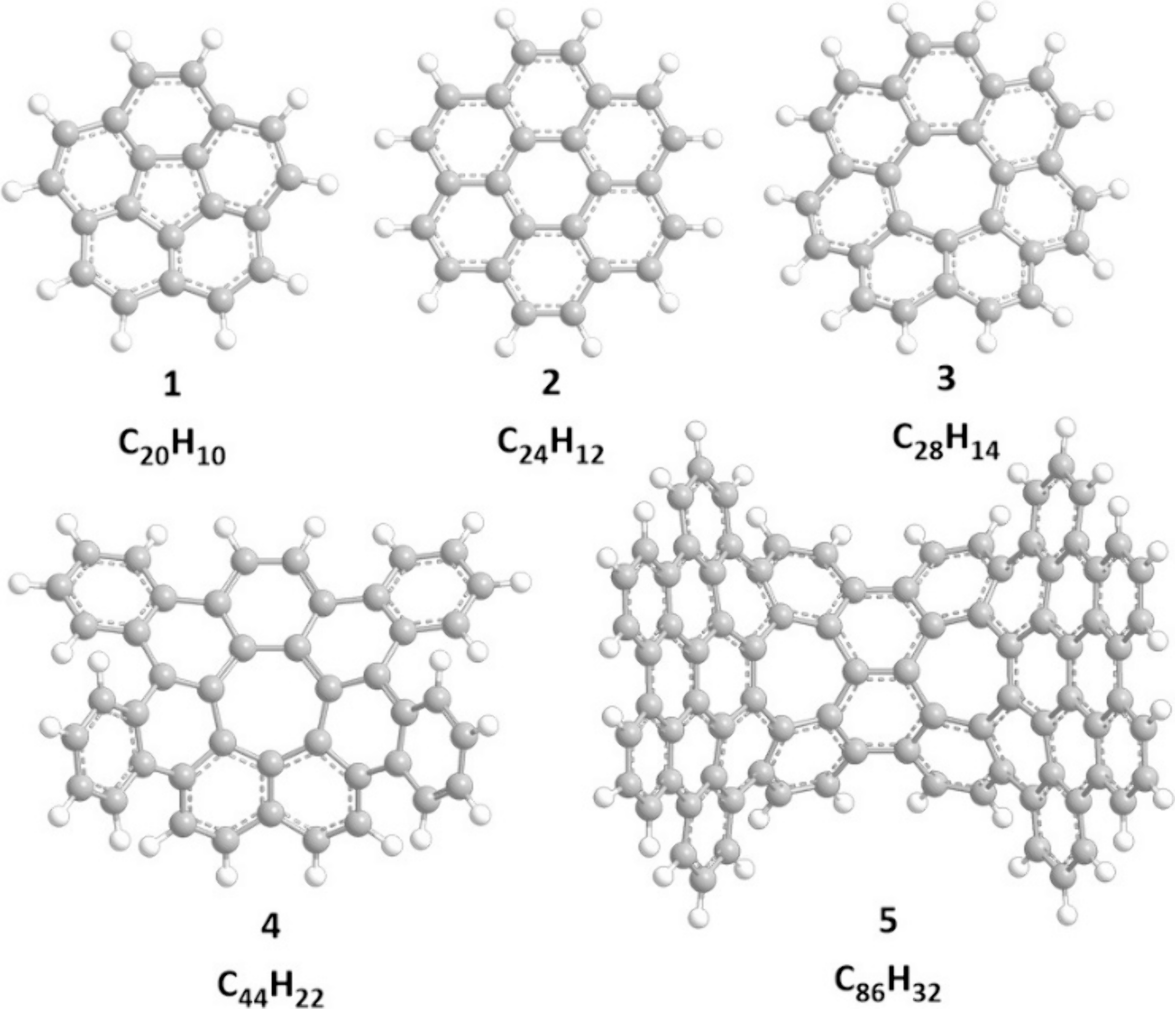
Representative bowl-shaped (**1**, C_20_H_10_, corannulene), planar (**2**, C_24_H_12_, coronene), and saddle-shaped PAHs (**3**, C_28_H_14_, [7]circulene) containing five-, six-, and seven-membered rings and more complex saddle-shaped structures with one (**4**, C_44_H_22_, tetrabenzo[7]circulene) or two seven-membered rings (**5**, C_86_H_32_).

Peri-fused planar PAHs such as 24π coronene (C_24_H_12_, **2**) (Figure 1) with solely six-membered (benzene) ring moieties are emphasized as molecular building blocks of zigzag nanoribbons^12^ and of fullertubes^13^ with zero Gaussian curvature.^14^ By embedding five-membered rings [cyclopentadiene (C_5_H_6_), fulveneallene (C_7_H_6_), fulvenallenyl (C_7_H_5_)] in the carbon backbone, molecular-bowl hydrocarbons and (fragments of) fullerenes (C_60_, C_70_) with positive curvatures are formed,^15^ with corannulene (C_20_H_10_, **1**) as the simplest prototype of a bowl-shaped hydrocarbon.^16^ In contrast, the introduction of seven-membered rings into a hexagonal planar network induces a negative curvature with a saddle shape.^5^ First prepared by Yamamoto et al. in 1983,^17^ [7]circulene (C_28_H_14_, **3**) has a central seven-membered ring surrounded with seven-fused benzenoid rings, representing a prototype saddle-shaped PAH and a fundamental building block of negatively curved nanostructures such as toroidal carbon nanotubes.^18^ The beauty of such saddle-shaped structures attracted extensive preparative interest, and more complex aromatic saddles embedded with seven-membered rings of tetrabenzo[7]circulene (C_44_H_22_, **4**)^19^ and *tert*-butylated derivative of negatively curved nanographene (C_86_H_32_, **5**)^20^ were prepared successively.

The distinct role of the azulene unit with a fused seven- and five-membered ring in molecular mass growth processes is reflected in the exotic, contorted saddle-shaped aromatic systems with both a positive and negative curvature of the carbon backbone^21−23^ along with the exceptional optoelectronic, magnetic, chemical, and quantum-coherent properties.^2,24−26^ These structures display higher fluorescence and larger HOMO–LUMO gaps,^5^ holding critical applications in the fields of photovoltaics^5^ and optoelectronic devices.^27^ Fundamental attention in traditional organic chemistry attracted the synthesis of azulene derivatives via, e.g., cycloaddition of 2*H*-cyclohepta-[b]furan-2-ones^28^ and fulvenes,^29^ Nozoe’s method,^30^ Scholl-type cyclization,^21,23^ and Pd-catalyzed [5+2] annulation.^31^ However, these pathways cannot rationalize the gas-phase molecular growth process to azulene alone in combustion systems and in the interstellar medium.

Here, by exploiting crossed molecular beam experiments along with electronic structure calculations and molecular dynamics (MD) simulations, persuasive evidence on the very first barrierless gas-phase formation of azulene via the bimolecular gas-phase reaction of the methylidyne radical (CH, X^2^Π) with indene (C_9_H_8_, X^1^A′) is offered. Importantly, by combining the explorative ability of ab initio molecular dynamics simulations with modern machine learning architecture, our results demonstrate that one of the largest *reactive* gas phase machine-learned surfaces in the field, CH + indene, can be trained, simulated, and rigorously tested. Moreover, this reaction exemplifies the prototype of a barrierless conversion of a six-membered ring to a seven-membered ring with the preparation of the azulene unit potentially initiating a starting point to saddle-shaped PAHs with seven-membered ring moieties in hydrocarbon-rich cold molecular clouds such as TMC-1. These findings dispute established paradigms that molecular mass growth processes to nonplanar PAHs, especially those containing seven-membered rings, operate only at high-temperature conditions such as in circumstellar envelopes of carbon stars or in combustion systems,^32^ proposing that the formation of the very first aromatic molecules with fused seven- and five-membered ring can be initiated via unconventional low-temperature chemistries in our Universe.

## Results

### Laboratory Frame

**Figure 2:**
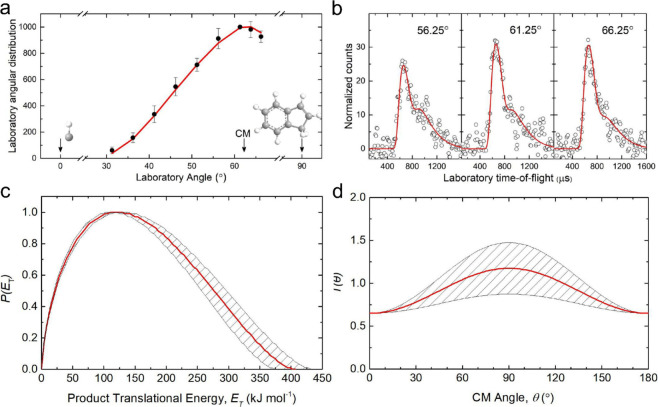
Laboratory data (LAD (a), TOF spectra (b)) and CM functions (CM translational energy (c), angular flux distributions (d)) of the reaction of methylidyne (CH) with indene (C_9_H_8_) at *m*/*z* = 128. The black circles with their error bars in the LAD indicate the normalized experimental distribution; the open circles in the TOF spectra delineate the experimental data. Carbon and hydrogen are color coded gray and white, respectively. The red lines delimit the best fit; shaded areas depict the error limits of the best fits.

The experiments were conducted under single collision conditions exploiting the crossed molecular beam approach.^33,34^ Bimolecular reactions of the methylidyne radical (CH, X^2^Π; 13 amu) with indene (C_9_H_8_, X^1^A′; 116 amu) resulted in reactive scattering signal detected at mass-to-charge (*m*/*z*) ratios of 128 (C_10_H_8_^+^/^13^CC_9_H_7_^+^) and 127 (C_10_H_7_^+^/^13^CC_9_H_6_^+^); the signal at 127 could be accumulated at a level of 51 ± 5% with respect to 128. No definite signal was observable at 129 *m*/*z*, indicating the absence of a C_10_H_9_ adduct. After scaling, the time-of-flight (TOF) spectra collected at *m*/*z* = 127 and 128 are superimposable, manifesting the existence of a methylidyne versus atomic hydrogen loss channel (reaction 1); the signal at *m*/*z* = 127 originates from dissociative ionization of the C_10_H_8_ product within the electron impact ionizer. TOF spectra were recorded at *m*/*z* = 128 at distinct laboratory angles between 30° and 67°, scaled, and integrated to ultimately extract the laboratory angular distribution (LAD) (Figure 2a). This LAD reveals a distribution maximum around the center-of-mass (CM) angle of 62.5 ± 0.7° and is spread over at least 35° within the scattering plane (Table S1). These findings indicate indirect scattering dynamics^35^ and the existence of chemically activated C_10_H_9_ complex(es), which decompose to the C_10_H_8_ product isomer(s) plus atomic hydrogen. Therefore, our experimental data alone reveal the atomic hydrogen displacement pathway to the C_10_H_8_ isomer(s) via reaction 1.

Since the atomic hydrogen can be eliminated from the methylidyne (CH) and/or the indene (C_9_H_8_) reactant(s) (reaction 2a–b), an experiment of D1-methylidyne (CD, X^2^Π; 14 amu) with indene was also conducted to extract the position(s) of the atomic hydrogen loss(es). Signals were collected at *m*/*z* = 129 (C_10_H_7_D^+^/^13^CC_9_H_8_^+^) and 128 (C_10_H_6_D^+^/C_10_H_8_^+^/^13^CC_9_H_7_^+^). Our experiments revealed identical TOF spectra at *m*/*z* = 128 and 129 with a ratio of 0.48 ± 0.03:1 also taking into consideration the ^13^C isotopic contributions. Note that the signal at *m*/*z* = 128 can also be connected to dissociative electron impact ionization of the C_10_H_7_D parent molecule. Within the error limits, the yields of the atomic hydrogen loss in the CH/C_9_H_8_ and CD/C_9_H_8_ systems are essentially identical, suggesting the gas phase formation of a species with the formula C_10_H_7_D (129 amu) and also the H loss from the indene (C_9_H_8_) reactant.
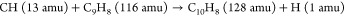
1
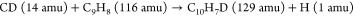
2a
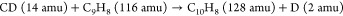
2b

### CM Frame

The laboratory data alone provide clear evidence of the formation of C_10_H_8_ isomer(s) via the methylidyne versus atomic hydrogen exchange in the bimolecular gas phase reaction of indene plus methylidyne radical; isotopic substitution experiments further verify that the H atom originates from the indene reactant. To expose the underlying reaction mechanism(s) and chemical reaction dynamics,^33^ the laboratory data are transformed to the center-of-mass (CM) reference frame. This transformation yields the center-of-mass translational energy, *P*(*E*_T_), and angular, *T*(θ), distributions (Figure 2c–d). Essentially, the laboratory data can be successfully reproduced with a single reaction channel, leading to C_10_H_8_ (128 amu) and H (1 amu) (reaction 1). The *P*(*E*_T_) distribution prolongs to a maximum energy release (*E*_max_) of 403 ± 29 kJ mol^–1^; these data can be exploited to recover the reaction energy of reaction 1, accounting for energy conservation via *E*_max_ = *E*_c_ – Δ_r_G, with the collision energy *E*_c_ and the reaction energy Δ_r_G for those products formed without internal excitation. Consequently, a reaction exoergicity of 381 ± 29 kJ mol^–1^ is derived. In addition, a distribution maximum of *P*(*E*_T_) of 120 ± 9 kJ mol^–1^ suggests a tight exit transition state associated with an extensive reorganization of the electron density when the reaction intermediate(s) decomposes unimolecularly to the final products. Furthermore, *T*(θ) depicts intensity over the complete angular range and is forward–backward symmetric. These findings are indicative of a long-lived reaction intermediate along with indirect scattering dynamics involving C_10_H_9_ complex(es) with lifetime(s) longer than the rotational period.^35^ Finally, the distribution maximum of *T*(θ) at 90° strongly infers geometrical constraints in the tight exit transition state, with an emission of the atomic hydrogen nearly perpendicularly to the rotation plane of the decomposing complex.^36^ These findings are also reflected in the flux contour map, which shows an overall image of the reaction and scattering process (Figure S1).

## Discussion

### Electronic Structure Calculations

**Figure 3:**
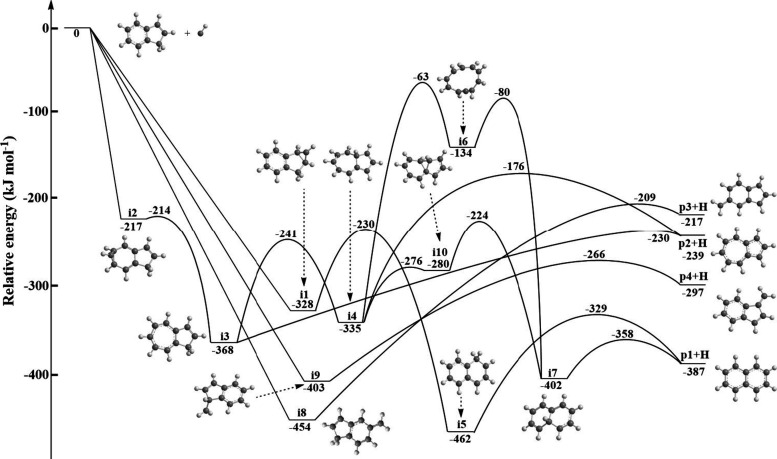
Potential energy surface (PES) for the reaction of methylidyne (CH) with indene (C_9_H_8_).

In the case of polyatomic reactions, it is often beneficial to merge crossed molecular beam experiments with electronic structure calculations, statistical calculations, and molecular dynamics simulations. First, the electronic structure calculations identified four C_10_H_8_ isomers (**p1**–**p4**) which can be eventually accessed through the unimolecular decomposition of ten C_10_H_9_ doublet radical intermediates (**i1**–**i10**) (Figure 3, Figure S2). A complete list of products which includes distinct structures with a CH_2_ moiety at different places is depicted in Figure S3 (Supporting Information). Accounting for the accuracy of ±5 kJ mol^–1^,^37,38^ a comparison of the computed reaction energies and the experimentally determined reaction exoergicity of 381 ± 29 kJ mol^–1^ suggests that at least the thermodynamically most stable naphthalene isomer (**p1**; ΔrG = −387 ± 5 kJ mol^–1^) is formed. However, the contributions of higher energy isomers including azulene (**p2**, −239 kJ mol^–1^), 5-methyleneindenyl (**p3**, −217 kJ mol^–1^), and 1-methyleneindenyl (**p4**, −297 kJ mol^–1^) cannot be excluded since these isomers could be hidden in the low-energy section of the translational energy distribution.

How is the reaction between indene (C_9_H_8_, X^1^A′) and the methylidyne radical (CH, X^2^Π) initiated? Methylidyne can either add to the carbon–carbon double bond or insert into a carbon–hydrogen single bond of the six- or five-membered ring, providing *defacto* four reaction mechanisms. The computations reveal that all these pathways are barrierless and lead to four initial reaction intermediates, **i1**, **i2**, **i8**, and **i9**. Note that distinct structures of **i1**, **i2**, **i8**, and **i9** can be formed via additions or insertions into different C–C and C–H bonds of the five- and six-membered ring. But the simplified PES is sufficient to describe the underlying reaction mechanisms (Supporting Information). Here, an addition of methylidyne to the carbon–carbon double bond of the five- or six-membered ring forms tricyclic intermediates **i1** or **i2**, which are stabilized by 328 or 217 kJ mol^–1^ relative to the separated reactants, respectively. Both intermediates **i1** and **i2** can subsequently isomerize via the ring-opening of the annulated three-membered cycle to singly hydrogenated naphthalene and azulene, namely the 1-hydronaphthyl radical (**i5**) and the 1-hydroazulenyl radical (**i3**). It should be noted that the methylidyne radical can add to six chemically inequivalent C–C bonds of the benzene moiety (Figure S4). All reaction intermediates **i2.1**–**i2.5**, which are bound by 203 to 217 kJ mol^–1^, eventually isomerize via ring opening to the same intermediate **i3**. Once prepared, **i3** can undergo facile unimolecular decomposition via atomic hydrogen loss from the CH_2_ moiety to **p2** (azulene, X^1^A_1_). Moreover, the migration of hydrogen from the CH_2_ moiety of **i3** to the bridging carbon may form **i4**. Three routes are open commencing with **i4**: (i) an energetically unfavorable ring opening via a significant barrier of 272 kJ mol^–1^ accessing a ten-membered ring intermediate **i6** followed by ring contraction to 4a-hydronaphthyl (**i7**), which can be considered as a hydrogenated naphthalene structure; (ii) a contraction of the seven-membered ring moiety of **i4** to a tricyclic intermediate **i10** carrying one six-, one three-, and one five-membered ring, and (iii) hydrogen atom loss from the bridging carbon to azulene (**p2**). The reaction sequences **i4** → **i6** → **i7** and **i4** → **i10** → **i7** are terminated by an emission of atomic hydrogen from the bridging carbon of **i7**, leading to the preparation of naphthalene (**p1**, X^1^A_g_). On the other hand, intermediate **i5** is connected to **p1** plus atomic hydrogen with a barrier of 58 kJ mol^–1^ relative to the separated products. Besides the addition pathways, the insertion of methylidyne into the C–H single bonds of the benzene or cyclopentadiene moieties leads to 5-methyleneindene (**i8**) or 1-methyleneindene (**i9**), respectively. In contrast to intermediates **i1** and **i2**, **i8** and **i9** are connected to **p3** and **p4** via simple insertion–elimination pathways. Which pathway dominates? Recall that the *T*(θ) depicts sideway scattering dynamics and hence a distribution maximum at 90°; this reveals an emission of the H atom nearly perpendicularly to the rotational plane of the decomposing complexes. This is supported by the computed geometries of the exit transition states leading to **p1** and **p2** via **i3** → **p2**, **i4** → **p2**, **i7** → **p1**, and **i5** → **p1** with the atomic hydrogen emitting at angles of 79.7°, 88.6°, 83.9°, and 81.9°, respectively, with respect to the rotation plane (Figure S5). We also utilized statistical RRKM theory to predict the branching ratios of C_10_H_8_ isomers formed within the limit of a complete energy randomization. Recall that the RRKM theory cannot predict the fraction of the initially formed reaction intermediates **i1** to **i4**. However, RRKM can be exploited to predict the branching ratios of **p1** to **p2** starting with any intermediate **i1** to **i4**. Here, the branching ratios depend strongly on the initial collision complex. At the experimental collision energy of 22 kJ mol^–1^, 95.3% of **i2** decomposes to **p2** with the remainingt 4.7% dissociating to **p1**. At the low collision energy of 0.5 kJ mol^–1^, **i2** is connected to **p2** and **p1** with ratios of 95.2% and 4.8%, respectively. At both conditions, the decomposition of **i1**, **i8**, and **i9** will only lead to **p1**, **p3**, and **p4**, respectively (Table S2).

Additional information about the underlying reaction dynamics can be provided from the D1-methylidyne (CD)–indene (C_9_H_8_) system. These isotopic substitution studies can be exploited to trace the origin of atomic hydrogen and/or deuterium elimination (Figure S2). If the reaction commences through addition of the D1-methylidyne radical (**i1** and **i2**), successive ring openings eventually incorporate the deuterium atom within a six- (**i5** and **i7**) or seven-membered ring (**i3** and **i4**). These intermediates are responsible for the formation of **p1** (D1-naphthalene) and **p2** (D1-azulene) plus atomic hydrogen, i.e., solely via atomic hydrogen loss, but not through emission of atomic deuterium. Should the reaction commence with insertion intermediate **i8** or **i9**, successive hydrogen losses yield D1-5-methyleneindenyl (**p3**) and D1-1-methyleneindenyl (**p4**). Therefore, the calculations reveal that only atomic hydrogen from the indene reactant can be eliminated in the pathways leading to **p1**–**p4**. This is well supported through the aforementioned experimental data of the CD–C_9_H_8_ versus CH–C_9_H_8_ system and the essentially identical ratios of *m*/*z* = 128 versus 129 (0.48 ± 0.03:1) and *m*/*z* = 127 versus 128 (0.51 ± 0.05:1), respectively.

### Reaction Dynamics Simulations

To tackle these ever-growing molecules with computational methods, ever-more efficient methods have been developed. For potential energy surface exploration, with the gold standard CCSD(T) method^39^ with extrapolation to the complete basis set (CBS) limit being the most trustworthy, systems with more than ten heavy atoms require compromises in accuracy made for the feasibility of the calculations.^40−42^ For dynamics simulations, even faster methods still must be used for the hundreds of thousands to millions of energy gradient calculations required. Here, the open question of the relative populations of the collision complexes resulting from the collisions between methylidyne radical (CH) and indene (C_9_H_8_) demands quasiclassical trajectory (QCT) simulations. QCT simulations offer theoretical understanding of the crossed molecular beam experiments of the methylidyne–indene system under experimental conditions. Ab initio molecular dynamics (AIMD) simulations at the experimental conditions are employed where trajectories commencing with a collision of the reactants are propagated by solving classical equations of motion with the energy gradients calculated with an ab initio method on-the-fly. However, AIMD simulations of the title reaction under interstellar conditions is infeasible. Under this condition, the collision energy between the reactants (scaled by their thermal temperature) is only 0.5 kJ mol^–1^. Considering that AIMD trajectories need to start with well-separated reactants for ergodic sampling (e.g., 10 Å in our simulation), it takes up to 11 ps for them to just collide with one another. Therefore, the time that it takes for the reactants to collide completely overwhelms the time that the vibrational energy redistribution of the intermediate takes. This is not only an unwise way to spend computational resources but also makes computation infeasible.

To overcome this issue, machine-learning molecular dynamics (MLMD) simulations are conducted to predict collision dynamics under interstellar conditions. Machine learning methods have been becoming more prominent for learning potential energy surfaces for their ability to reproduce arbitrarily high-level ab initio energies at a fraction of the cost.^43−45^ Our group has reported that the ML potential should be validated beyond what is normally accepted as “chemical accuracy”, which is obtained from analyzing its accuracy on a preselected set of geometries. Here the cross sections of intermediates obtained from AIMD simulations at the experimental conditions are used to validate the ML potential (Supporting Information, Tables S3–5, Figure S6). Since the excess energy at the experimental conditions (22 kJ mol^–1^ of the separated reactants) is much larger than it is at the interstellar conditions (0.5 kJ mol^–1^ of the separated reactants), the relevant chemical spaces sampled in the former is larger than in the latter. Therefore, an ML potential that is deemed trustworthy to predict the population of intermediate at the experimental conditions is also trustworthy at the interstellar conditions, and further validation is not necessary.

**Figure 4:**
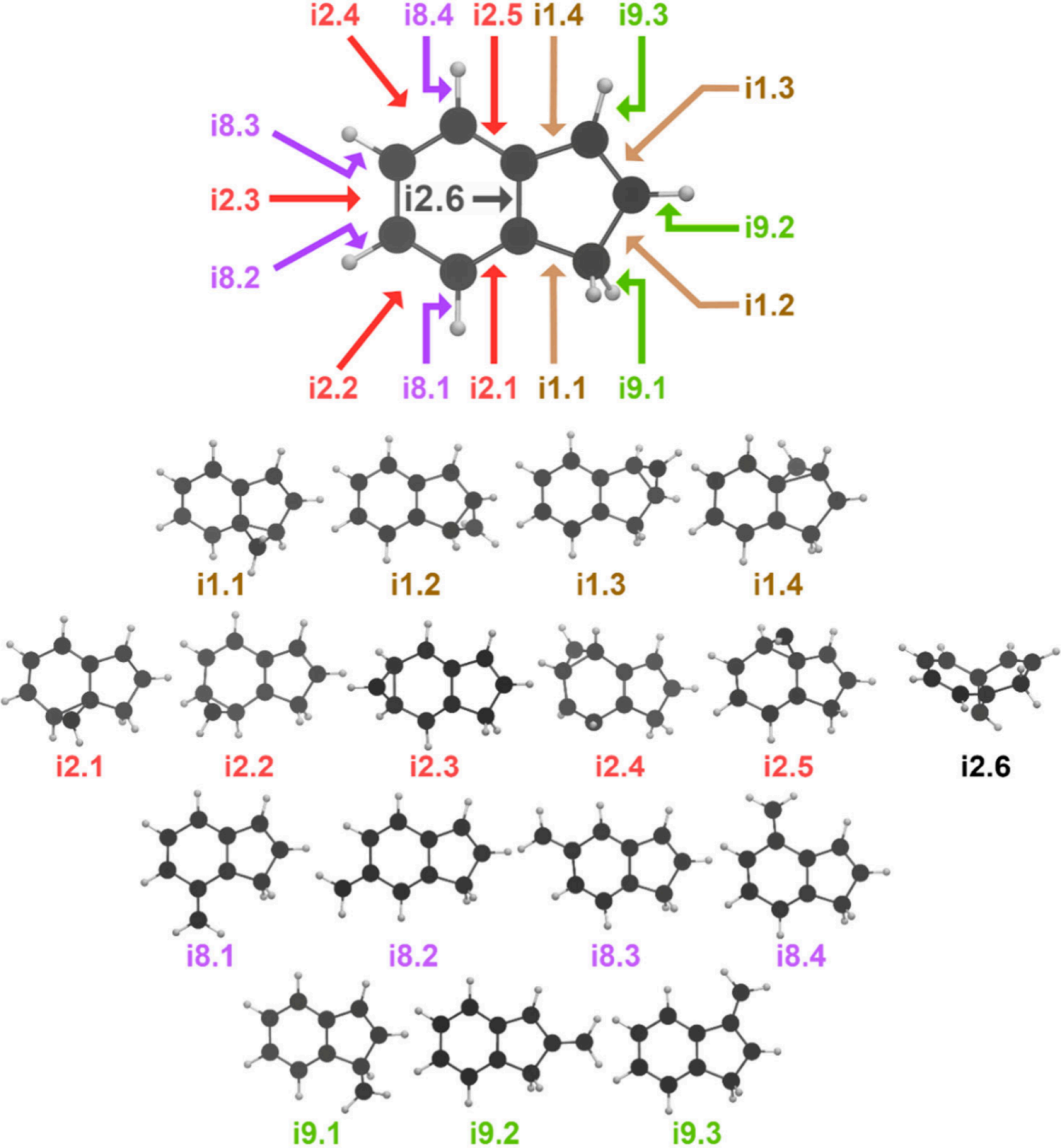
Initial collision-induced intermediates found in AIMD simulations at experimental condition. **i1** and **i2** are formed via the addition of a CH radical to a C–C bond of the five- and six-membered rings, and **i8** and **i9** are formed through the insertion of a CH radical in a C–H bond of the five- and six-membered rings of indene (C_9_H_8_).

In the simulations at the experimental condition, out of the 700 AIMD trajectories, 296 are nonreactive, 8 form CH_2_ plus indenyl via hydrogen atom abstraction (a representative animation is shown in Video S1), and 396 remain in molecular complexes (undissociated) after the first 500 fs. These phenomena align with the aforementioned experimental results that the reaction involves a long-lived intermediate. Aside from the CH_2_–forming trajectories, all reactive trajectories start by either addition to a C–C bond or insertion into a C–H bond. Additions to the ten C–C bonds of the five- and six-membered ring form **i1.x** and **i2.x**, while insertions to the eight C–H bonds in the five- and six-membered ring form **i9.x** and **i8.x** (**x** indicates addition or insertion to different C–C or C–H bonds), respectively (Figure 4). The AIMD simulations show that additions are preferred over insertions at a ratio of about 3:1, highlighting the favorable cone of acceptance of the π electronic system. However, the relative population of **i2.x** quickly decreases from 41.6% to 1.9% within the first 500 fs due to the very low isomerization barriers (3 kJ mol^–1^) of **i2.x** → **i3** (Figure S7, Table S5). For comparison, the isomerization barrier of the addition intermediates **i1.x** to **i5** is significantly higher (98 kJ mol^–1^), and thus its population declines much slower (from 23.6% to 19.7% within the same amount of time). Intermediates formed through insertion (**i8.x** and **i9.x**), lying around 237 and 106 kJ mol^–1^ below the corresponding separate products **p3** and **p4** plus H, are highly stable. Therefore, their populations did not change significantly within the first 500 fs. The further breakdown of these entrance channel intermediates, including minor isomerization pathways, can be found in Figure S8.

The simulations show that the three C–C bonds on the six-membered ring furthest from the central C–C bond possess the largest reactivity (Figure S8, formation of **i2.2**, **i2.3**, and **i2.4**). For the **i1.x** isomers, the addition to the C–C bond opposite the sterically hindering CH_2_ group (formation of **i1.3**) is preferred over addition to the other bonds (**i1.1**, **i1.2**, **i1.4**). Addition to a C–C bond next to the CH_2_ group added CH to form either **i1.1** or **i1.2**. In contrast, insertion into a C–H bond on the six-membered ring is relatively nonselective. On the five-membered ring, insertion is preferred to one of the C–H bonds on the CH_2_ group. While the predominant mechanism is to isomerize from **i1.x** to **i5** and from **i2.x** to **i3**, a few trace intermediates produced through alternative pathways have been observed. For example, some trajectories from **i1.3** proceed via the C–C bond rupture of the five-membered ring forming **i11** (Figures S9–10, Video S2), for which the simulations reveal no further isomerization within the first 500 fs. All insertion pathways (forming **i8.x** or **i9.x**) do not isomerize further within the simulation time.

Overall, the populations of the entrance channel intermediates become stable within 500 fs after collision, indicating this length of time is long enough for sufficient vibrational energy redistribution, and the vibrational energies are thermalized between different vibrational modes. The same behavior was reported for the methylidyne–vinylacetylene collision.^46^ As shown in Figure S7, the cross sections predicted by MLMD under experimental conditions are within the error bars of their AIMD counterpart under the same condition. Here we emphasize that minimizing the differences in dynamics between MLMD and AIMD under a known condition that has larger excess energy is essential to extending MLMD to other conditions with lower excess energy. This remarkable level of accuracy of MLMD speaks to the accuracy and power of the ML potential.^47^ The populations of the entrance channel intermediates under interstellar conditions can be found in Figure S11, which largely resembles the populations found under experimental conditions. The statistical differences lie in the population of **i2.x** (and **i3**), which is more dominant under interstellar conditions, and **i9.x**, which is less dominant under interstellar conditions. All of the remaining intermediates are grouped into **iO**. The populations of **i1**, **i2**, **i3**, **i5**, **i8**, and **i9** by the end of the MD simulations (0.5 ps) were tabulated in Table S5. These populations were combined with the results of RRKM calculations to evaluate the MD-corrected product branching ratios, resulting in 29.4% (**p1**), 56.7% (**p2**), 6.8% (**p3**), and 7.1% (**p4**) at a collision energy of 0.5 kJ mol^–1^ and 29.2% (**p1**), 45.7% (**p2**), 8.9% (**p3**), and 16.2% (**p4**) at a collision energy of 22 kJ mol^–1^ (Table S2). These results reveal that azulene (**p2**) represents the most probable product in the methylidyne–indene reaction under both experimental and interstellar conditions, with fractions of up to 60% with about 29% accounting for the naphthalene isomer at 10 K. Therefore, the results from the MD simulations reinforced the predictions of the experimental determined products: azulene and naphthalene.

The facile conversion of a six-membered ring to a seven-membered ring, especially the barrierless route connecting indene (C_9_H_8_) with azulene (C_10_H_8_), represents the missing link between small carbon clusters and carbonaceous nanoparticles in combustion chemistry and astrochemistry. First, the experimentally verified pathways to azulene under combustion conditions has been long-pursued since this might be the initial step to azulene-embedded nanographene and saddle-shaped nanostructures,^2,48−50^ broadening our understanding of the origin and evolution of the combustion soot particles.^51,52^ Second, ring molecules including cyclopentadiene (C_5_H_6_),^9^ cyano-substituted benzene (C_6_H_5_CN)^53^ and naphthalene (C_10_H_7_CN),^54^ and indene (C_9_H_8_)^9^ have been revealed to be widespread in cold molecular cloud of TMC-1 with the observed fractional abundance relative to H_2_ of up to (1.6 ± 0.3) × 10^–9^. Only recently, 1-cyanopyrene, a four-ring PAH, is detected in TMC-1, suggesting that the carbon supplied to young planetary systems might be carried by PAHs originated in cold molecular cloud.^55^ However, less attention has been devoted to the search of the seven-membered rings. Here, our investigations of the low-temperature gas-phase formation of azulene from indene might provide direct connections between the five- and six- membered ring chemistry to the seven-membered ring chemistry, which could open up new routes in studying bottom-up reaction network in cold molecular cloud.

## Conclusion

Our combined crossed molecular beam, electronic structure calculations, and molecular dynamics simulations provided compelling evidence on the very first gas-phase barrierless preparation of azulene (C_10_H_8_, X^1^A_1_), the prototype nonbenzenoid PAH with fused seven- and five-membered ring, under single collision conditions via the elementary reaction of the simplest organic radical, methylidyne radical (CH), with indene (C_9_H_8_). MD-corrected RRKM computations revealed that azulene is formed predominantly (56.7%) under low-temperature conditions of TMC-1 and naphthalene is formed with a branching ratio of 29.4%. This facile and barrierless gas-phase pathway to azulene under single collision conditions signifies a new and fundamental starting point for saddle-shaped PAHs and carbonaceous nanostructures in TMC-1 inflicted by the seven-membered ring. This exploration is also appealing from the viewpoint of physical organic chemistry in which the efficient ring expansion from six- to seven-membered ring with the formation of an azulene unit is still not fully understood. Moreover, the excellent performance of the derived machine-learned molecular dynamics (MLMD) approach in predicting the collision dynamics under low-temperature interstellar conditions reveals that crossed molecular beam studies augmented with MLMD simulations have advanced to a new level such that complex, polyatomic reactions involving bicyclic PAHs relevant to the interstellar environment can be explored at the microscopic, molecular level.

Although the azulene molecule has not been identified, considering the high abundance of CH radical ((11 ± 5) × 10^–9^)^56^ and indene molecule ((1.6 ± 0.3) × 10^–9^)^9^ in TMC-1 and its large dipole moment of 0.80–1.08 D,^57,58^ azulene is a promising candidate for the future astronomical observations. Furthermore, considering that cyanoindene (C_9_H_7_CN) has been identified in TMC-1 with an abundance of (1–3) × 10^–11^ relative to H_2_,^59^ and that ethynylindene (C_9_H_7_CCH) represents a promising candidate for the astronomical observations,^60^ the low-temperature gas phase route to highly reactive azulene also represents a universal template toward the formation of ethynyl (C_2_H) or cyano (CN)-substituted azulene. In TMC-1, due to the low density, only two-body collisions are relevant. Overall, our investigations might represent a very first step to provide critical constraints on the largely elusive molecular mass growth process for aromatics, especially the saddle-shaped PAHs under low-temperature conditions of TMC-1.

## Supporting Information Available

Supplementary discussion, supplementary MLMD details, experimental and computational methods, experimental parameters (Table S1), RRKM predictions (Table S2), molecular dynamics simulations details (Tables S3–5), optimized coordinates and calculated vibrational frequencies for stationary structures (Table S6), flux contour map (Figure S1), PES for the CD-C_9_H_8_ reaction (Figure S2), supplementary computational results (Figures S3–5, Figure S9), supplementary molecular dynamics simulations results (Figures S6–8, Figures S10–11) (PDF)

## Notes

Simulations at the experimental conditions.
