# Supplementary material for: Directed Gas-Phase Formation of Azulene (C10H8): Unraveling the Bottom-Up Chemistry of Saddle-Shaped Aromatics
Source: ACS Cent Sci. 2025 Feb 4;11(2):322–30. doi: 10.1021/acscentsci.4c01606 (PMC11869134; doi:10.1021/acscentsci.4c01606)
Supplement: Supplementary file 1 — oc4c01606_si_001.pdf [file oc4c01606_si_001.pdf]

Directed Gas-Phase Formation of Azulene ($C_{10}H_8$): Unraveling the Bottom-Up Chemistry of Saddle-Shaped Aromatics

Zhenghai Yang[a†], Kazuumi Fujioka[a†], Galiya R. Galimova[b†], Iakov A. Medvedkov[a], Shane J. Goettl[a], Rui Sun[a*], Alexander M. Mebel[b*], Ralf I. Kaiser[a*]

[a] *Department of Chemistry, University of Hawai'i at Manoa, Honolulu, Hawaii 96822, USA*

[b] *Department of Chemistry and Biochemistry, Florida International University, Miami, Florida 33199, USA*

*Email: ralfk@hawaii.edu; ruisun@hawaii.edu; mebela@fiu.edu;

Supplementary discussion

As shown in Figure 4, Additions to the ten C-C bonds of the five- and six-membered ring form **i1.x** and **i2.x**, while insertions to the eight C-H bonds in the five- and six-membered ring form **i9.x** and **i8.x** (x indicates addition or insertion to different C-C or C-H bonds), respectively. First, for the **i1.x** isomers, the simulations show that addition to the C-C bond opposite of the sterically hindering CH_2 group is preferred. And **i1.x** structures can only isomerize via the ring-opening of the annulated three-membered cycles to singly hydrogenated naphthalene (**i5**) which is then connected solely to naphthalene (**p1**). Second, the addition of CH to chemically inequivalent C-C bonds of the benzene moiety leads to **i2.1**-**i2.5**, which are bound by 203 to 217 kJ mol^{-1}, eventually isomerizing via ring opening to the same intermediate **i3**. Third, the insertions of CH to the eight C-H bonds in the five- and six-membered rings lead to seven different intermediates (**i8.1**-**i8.4**, **i9.1**-**i9.3**) which can form four different products (Figure S3) through simple insertion-elimination pathways. Considering that **p3** and **p4** only represent the minor products and the formation mechanisms are similar, only **p3.1** and **p4.1** is included in the simplified PES. In summary, our simplified PES which include only the most probable structures of **i1**, **i2**, **i8**, and **i9** is sufficient to explain the underlying reaction mechanisms in the CH – C_9H_8 reactions.

Supplementary MLMD details

It is extremely challenging to simulate the reaction at interstellar conditions (10K) since the reactive cross sections reaching any collision complex increases as the collision energy decreases. In the extreme case where the collision energy approaches zero, the cross section approaches infinity as the attractive potential energy takes over the dynamics of the reaction. From the perspective of dynamics simulation, this means that due to a larger maximum impact parameter (b_{max}) and longer trajectories need to be simulated. Here, the Recursively Embedded Atom Neural Network (REANN) is utilized for the first time to train a machine-learned PES (MLPES) in bimolecular reactions under low-temperature interstellar conditions (10K). The derived machine-learned molecular dynamics (MLMD) depicted excellent performance in predicting the collision dynamics.

The AIMD simulations at the experimental condition (collision energy of 22 kJ mol^{-1}) provides the training and validation sets for the REANN ML potential. Due to the millions of geometries visited in the trajectories, some metric should be employed to refine them and generate a training set of manageable size. In practice, this is difficult to do. For instance, it may be reasonable to use a traditional distance matrix deviation (DMD) as the metric, which is defined for two configurations A and B as the minimum RMSE between DM(P(A)) and DM(B) over all permutations of indistinguishable atoms (Eq. 1).[x] For this system, going through all permutations for 10 carbon atoms and 9 hydrogen atoms ((10!)(9!) > 10^{12} permutations) is infeasible thus only a few following chemical intuition are attempted.

$$DM(A) = \left[r_{H1,H2}^{-2}, r_{H1,H3}^{-2}, r_{H1,H4}^{-2}, \ldots, r_{H1,C10}^{-2}, r_{H2,H3}^{-2}, \ldots, r_{C9,C10}^{-2}\right] \quad (1)$$

Here we adopt the DMD (0.01 and 0.05 A^{-2} for reactive and non-reactive trajectories) to generate the training and validation sets from AIMD trajectories. The population of geometry in the close vicinity of each isomer in the training and validation sets are summarized in Table S3-4 and Figure S6.

After generating the training and validation sets, a number of models are trained with different hyperparameters (Table S3-4). For the chosen model, the absolute errors (MAEs) and root mean square errors (RMSEs) of the energies and forces of the dynamics simulations of the CH – C_9H_8

reaction are 4.5 kJ mol^{-1}, 11.7 kJ mol^{-1}, 1.8 kJ mol^{-1} $Å^{-1}$, and 3.9 kJ mol^{-1} $Å^{-1}$, respectively, which is larger than what has been considered as "chemically accurate" by the ML community.[1] It is important to note that ML potentials with small energy and force errors ("static errors") do not necessarily result in accurate MLMD considering that the validation set employed in generating those static errors are insufficient in representing the chemical space of the actual reaction dynamics. As shown in a recent study, a more rigorous benchmarking of the MLMD involves information on the dynamics of the reaction such as the cross section, scattering angles, etc. ("dynamical errors").[2] Therefore, the AIMD cross section of the intermediates at the experimental condition are used to validate the MLMD before applying the latter to the reaction at the interstellar condition. The PES of the reaction (Figure 3) features highly stable intermediates, e.g., 328 and 217 kJ mol^{-1} lower than the separated reactants for **i1** and **i2**, respectively. Previous research has shown that the decomposition of these low-lying intermediates is beyond the time scale of AIMD simulations of bimolecular collisions under similar conditions [3]. Nonetheless, AIMD simulations are still informative as they characterize the evolution of various collision-induced intermediates. When these intermediates are first formed, certain vibrational modes are highly excited and frequent isomerization are observed. The relative population of intermediates becomes stable after sufficient intramolecular vibrational energy redistribution (IVR), which can then be coupled with RRKM theory to predict the overall product branching ratio of the reaction.

In the simulations, the maximum impact parameter (b_{max}) found from AIMD is b = 7.0 Å; and out of the 700 AIMD trajectories, 296 are non-reactive, 8 form CH_2 (a representative animation is shown in (Video S1), and 396 remains in the molecular complex (undissociated) after first 500 fs. AIMD simulations with a finite amount of trajectories always predict the cross sections of the intermediates with uncertainty. Therefore, even accurate MLMD should not be expected to match the cross sections from AIMD, but instead, falls within the standard deviation (error bars) of AIMD simulation. Reproducing the dynamics observed in an ensemble of AIMD trajectories is a much higher standard than the commonly-adapted static errors in energy and energy gradients.

Supplementary ML potential details

Our ML potential is trained from the *ab initio* calculations and possesses certain desired properties (e.g., smoothness, size consistency, and energy extensivity). It is a function (f) that projects the geometry of a molecule consisting of N atoms (q, dimension: $3N$) to its potential energy (U, dimension 1), i.e., $f(q) = U$. The potential energy gradient of the system (g, dimension: $3N$) is obtained numerically from U. The excess amount of energy available to the system (E') is an independent variable that is neither an input nor an output of the function f.

It should also be made clear that the ML potential does NOT directly predict any of the observables of the bimolecular collision, including relative populations of species, branching ratio, etc. If it was, then we agree with the reviewer that our ML potential, validated at a high excess energy (e.g., experimental condition), should not extrapolate to a low excess energy (e.g., interstellar condition). Instead, energy gradients obtained from the ML potential are used to propagate the coordinate of the system, which is commonly known as molecular dynamics (MD) simulations. Here we attached to representation MD trajectories in Figure S12, where the potential energy fluctuates as the coordinate of the system evolves with time.

The next question then becomes how much of the geometrical space (i.e., the range of q) the ML potential (i.e., a function $f(q) = U$) can cover with controlled accuracy. If the entire geometrical space (i.e., all possible value of q) was accurately learned, then it would work for any conditions, including the experimental conditions and the interstellar conditions that are of interest. However, we would like to point out to the reviewer that no ML potential has ever made this claim. Training an entire geometrical space would entail learning unphysical, high-energy regions of the surface with nuclei either unphysically close to each other or molecules completely ripped apart into atoms; not only would learning these regions be exceedingly difficult but they would not add any useful information for a physically relevant simulations, which is the goal of this (and any other reasonable) reaction dynamics simulation. Instead of the entire geometrical space, the most common approach is to learn the energetically accessible portion of the geometrical space, namely: given some reference energy (e.g., the energy of the reactants, $U_0 = f(q_{reactants})$) and an excess energy (e.g., the rotational, vibrational, and translational excitation of the reactants, $E' = E'_{rot} + E'_{vib} + E'_{trans}$), the portion of the geometrical space possessing potential energy that is lower than total energy available (E) in the system (i.e., all q such that $f(q) < E = U_0 + E'$).

Regarding the current study, we take a modest yet reasonable undertaking of learning the geometrical space corresponding to the bimolecular association of the reactants and the first few intermediates (defined in the Methods as those up to 0.5 ps after the collision) – we name this geometrical space the entrance channel of the reaction. If our ML potential has accurately learned the energetically accessible portion of the entrance channel under the experimental conditions, then it would also have learned the energetically accessible portion of the geometrical space under the interstellar conditions, as the former encompasses the latter (see Figure 13). This statement should be axiomatic, as there is more excess energy under experimental conditions than the interstellar conditions. As one finds in graduate level physical chemistry textbooks, the number of states in the phase space, which includes both the geometrical space and the momentum space, scales exponentially with the excess energy.[4-6] In fact, as a proof of concept, we conducted unimolecular *ab initio* molecular dynamics (AIMD) simulations from more than 20 intermediates relevant to the entrance channel under interstellar conditions. More than 300,000 geometries sampled in these trajectories are compared to the original training set, which contains more than 161,000 geometries and was gathered from AIMD simulations under experimental conditions. Only 35 geometries out of the 300,000 geometries were deemed "novel" compared to the original training set. This test proved that the energetically accessible geometrical space under experimental conditions encompasses its counterpart under interstellar conditions to a negligible statistical error.

Then the ultimate question becomes whether the ML potential is properly trained for the energetically accessible portion of the entrance channel under the experimental condition. This is the purpose of the benchmarking reported in the main test and elaborated more in the SI. If the reviewer was concerned about the accuracy of the ML PES for individual trajectories rather than over the entire training set, we overlay in Figure S12 the error of the ML predicted energy for two representative trajectories, to hopefully allay some of these concerns. We note that error analysis is of great interest to us, so we did not just train a model to lower the static errors (i.e., the deviation in the energy and energy gradient between the *ab initio* potential and the ML potential), but also chose a model that had acceptable dynamics errors (Table S3). The populations of relevant intermediates in the entrance channel over time (Figure S7) demonstrate good agreement with AIMD simulations. In fact, over this time period, the species populations largely stabilize which

suggests no large number of new geometries would be missed if sampling was extended over more of simulation.

Methods

Experimental Methods

The gas-phase reaction of methylidyne (CH, $X^2\Pi$) with indene (C_9H_8, X^1A') was performed in a crossed molecular beam apparatus utilizing mass spectrometric (MS) detection in time-of-flight mode and a beam crossing angle of 90°.[7] In brief, two supersonic beams of reactants collide in the interaction region of a large scattering chamber with the existence of an oxygen-free high-conductivity copper cold shield cooled to 10 K (CTI Cryogenics, 1020) to reduce the background from the straight-through species. During the experiment, the supersonic beam of methylidyne was created through photodissociation (COMPex 110; KrF) of helium seeded bromoform ($CHBr_3$; Aldrich; ≥ 99 %) at 283 K with a backing pressure of 2.2 atm.[8] The 248 nm output was focused 1mm downstream the nozzle. Under single collision conditions, the CH beam is rotationally cooled to 14 ± 1 K via supersonic expansion and characterized by laser induced fluorescence technique.[9,10] After the skimmer and chopper wheel, the selected CH beam was defined with a v_p (peak velocity) of 1897 ± 31 m s^{-1} and an S (speed ratio) of 12.2 ± 0.7 (Table S1). The second supersonic beam was generated by expanding krypton (Praxair, 99.999%) seeded indene (C_9H_8) (TCI) at 550 torr backing pressure and room temperature resulting in a peak velocity of 416 ± 15 m s^{-1} and speed ratio of 10.2 ± 0.8. Both beams were released by Proch-Trickl piezoelectric pulse valves operated at -400 V with opening times of 80 μs; the pulse valves and the laser were modulated at 60 and 30 Hz, respectively, for a laser on minus laser off background subtraction. Bromoform-d ($CDBr_3$; Aldrich) was also used as a precursor yielding a D1-methylidyne beam (CD, v_p:1882 ± 29 m s^{-1}, S:12.3 ± 0.8). Both primary and secondary reactants were held in a stainless-steel bubbler. Finally, the collision energy E_c and laboratory CM angle θ_{CM} in the CH – C_9H_8 system are determined to be 22.1 ± 0.8 kJ mol^{-1} and 62.5 ± 0.7°, respectively (Table S1). A triply differentially pumped, and rotatable detector equipped with an electron impact ionizer, a quadrupole mass filter, and a Daly type ion detector, was utilized to collect the TOF spectra at desired angles which reflect the velocity and angular distributions of the isomer products formed in the reaction.[11,12] The detector

was operated under liquid nitrogen-cooled ultrahigh vacuum conditions of around 7×10^{-12} torr. To interpret the underlying physics of the scattering data and derive the quantitative information on the reaction dynamics, a forward convolution method was employed to fit the data from the laboratory frame to the CM frame. The total CM product flux $I(u, \theta)$, in which u, θ represent the CM product velocity and scattering angle, was analyzed to derive the reaction dynamics information of the reactive scattering process. $I(u, \theta)$ can be conveniently separated into the $P(E_T)$, and the $T(\theta)$ flux distributions, namely $I(u, \theta) \approx P(u) \times T(\theta)$, and the $T(\theta)$ and $P(E_T)$ functions were varied iteratively until a best fit of the laboratory TOF spectra and angular distribution is obtained.[13]

Electronic structure calculations

Geometric structures of various species (CH and C_9H_8, different products, $C_{10}H_9$ intermediates and transition states) on the PES accessed by the reaction of methylidyne radical with indene were optimized at the hybrid DFT B3LYP level with the 6-311G(d,p) basis set.[14,15] Vibrational frequencies calculations were carried out using the same B3LYP/6-311G(d,p) method. Single-point energies were further refined using the G3(MP2,CC)//B3LYP/6-311G(d,p) variant of the G3 model chemistry scheme.[16-18] The anticipated accuracy of the composite approach relative energies is typically within 5-10 kJ mol^{-1} or better.[18] According to our experience in calculating PES for large systems involving complex ring molecules, especially the PAHs, the G3(MP2,CC) composite scheme still represents one of the most optimal methods in terms of the balance of accuracy and efficiency. The derived accuracy, typically within 5-10 kJ mol^{-1} or better, is sufficient to predict the formed products in the experiment. Explicitly-correlated CCSD(T)-F12 are likely to improve the anticipated accuracy but they are significantly more expensive. Moreover, our recent comparisons of the G3(MP2,CC) and CCSD(T)-F12 relative energies for intermediates and transitions states for similar systems have shown a close agreement (within 4-5 kJ mol^{-1} or less) between the two methods.[19] The GAUSSIAN 09[20] and MOLPRO 2010[21] software packages were utilized for the ab initio calculations. For unimolecular reaction steps on the $C_{10}H_9$ PES, which follow initial association of CH with indene, rate constants were assessed within the framework of Rice-Ramsperger-Kassel-Marcus (RRKM) theory.[22-24] In the RRKM calculations of energy-dependent rate constants, the internal energy of each species (intermediate or transition state) was assumed to be equal to a sum of the collision energy and the energy of chemical

activation, which in turn equates to a negative of the relative energy of the species with regard to the CH + C_9H_8 reactants. The zero-pressure approach is justified by the fact that the reacting $C_{10}H_9$ intermediates in crossed molecular beams cannot undergo any collisional activation/deactivation. Next, first-order kinetic equations were solved in steady-state approximation[25] using the computed RRKM rate constants and employing our own Unimol code, to obtain product branching ratios under single-collision conditions.[26]

It should be noted that the $C_{10}H_9$ PES has been thoroughly explored in previous theoretical studies in relation to other reactions. For instance, few detailed accounts[27,28] show that even isomers containing only one ring (six- or five-membered) reside significantly higher in energy than two-ring structures. Thus, the one- and two-ring opening pathways in the CH + indene reaction are highly unfavorable and non-competitive, nor they were observed in the MD simulations of the reaction entrance channels.

Molecular dynamics simulations

Quasiclassical trajectories (QCT) were simulated with *ab initio* molecular dynamics (AIMD) using VENUS/NWChem (version 6.8).[29-31] Trajectories used a velocity Verlet integrator with a 0.5 fs time step, which was lowered if the trajectory experienced a greater than 4.2 kJ mol^{-1} (1.0 kcal mol^{-1}) energy jump or drift in total energy. Benchmarking from previous AIMD studies on similar reactions (e.g., CH + H_3CCCCH_3 and CH + $H_2CCHCCH$ reactions[3,32]) under likewise conditions have shown that, compared to other computationally efficient *ab initio* methods, B3LYP[33] has relatively low errors in reproducing energies of reference CCSD(T)[34] intermediates with numerically stable trajectories. Thus, the B3LYP/6-31G* method was used in this study.[35] Initial coordinates and momenta of the atoms in the system were sampled to mimic experimental conditions: the CH is set at its ground vibrational and rotational state; the indene is sampled from a thermal distribution of 10 K; the two reactants are separated by 10 Å (center of mass distance) and given a collision energy of 22 kJ mol^{-1}. The ML potential selected for dynamics simulations used 16 radial Gaussian functions (*Nwave* = 16), a cutoff of 10 Å, 2 hidden layers with 128 features each, a single residual block (*Nblock* = 1) and a maximum angular momentum of 2 (*Nipsin* = 2), resulting in 469,058 parameters; details on its creation are supplied below. 100 trajectories were simulated at impact parameters of b = 1, 2, 3, ... Å until b_{max}, the impact parameter at which no

reactive trajectories are found. For each intermediate of interest, their cross sections were obtained as:

$$\sigma = \int P(b)b\,db \approx \sum_{i=1} P(b_i)b_i\,\Delta b$$

where P(b) is the fraction of trajectories of which the reactive intermediate of interest is formed.

Simulating the reaction at near interstellar conditions (temperature = 10 K) is extremely challenging as the cross section of the intermediate increases as the collision energy decreases. In the extreme case where the collision energy approaches zero, the cross section approaches infinity as the attractive potential energy takes over the dynamics of the reaction. From the perspective of dynamics simulation, it means that more (due to large b_{max}) and longer (the molecules take more time to collide) trajectories need to be simulated. Carrying out such a simulation with AIMD is not feasible for this project. Therefore, a machine-learned (ML) potential energy surface (PES) is developed with the capability of quickly predicting the energy and energy gradients for the dynamics simulation. Typically, developing a MLPES requires a diverse set of geometries of the molecule of interest and their energies/gradients, which is divided into a training and a validation set. A certain machine-learning model is chosen to develop MLPES by learning from the training set and its performance is assessed by the deviation between the energies/gradients from the MLPES and *ab initio* methods in the validation set. This type of deviation is referred to as static error, as it is generated with a number of static geometries. Previous studies have shown that a MLPES with small static energy does not necessarily lead to accurate reaction dynamics obtained from AIMD simulations. This is due to the chaotic nature of the trajectory, which could traverse through geometries that are not represented in the training and validation set.[2] Therefore, dynamics error, defined as the deviation between trajectory ensemble average properties (e.g., product branching ratio, relative translation energy of the products, etc.) generated from MLPES dynamics and AIMD dynamics should be used in assessing the performance of MLPES. The training and validation sets were obtained from the 700 AIMD trajectories carried out at the experimental conditions, of which 56% formed long-lived molecular complexes. These AIMD trajectories consist of millions of geometries (and their corresponding energies and gradients), which were filtered through to eliminate redundancy according to inverse pairwise atomic distances (or 'distance matrices') for the efficient of training.[36,37] The training and validation sets contain

121,170 and 40,390 distinct geometries, respectively, ranging over nearly 150 kcal/mol with a detailed breakdown in the Supporting Information.

The MLPES is trained using the Recursively Embedded Atom Neural Network (REANN) software.[38] The machine-learned molecular dynamics (MLMD) makes use of the Atomic Simulation Environment (ASE) package.[39] Multiple MLPES (trained with different parameters, such as radial Gaussian functions (*Nwave*), atomic cutoffs, number of features per layer, number of residual blocks (*Nblock*), maximum angular momentums (*Nipsin*), and random seeds) are made; 8 MLPES with reasonable force and energy errors are given more training time and ultimately tested with MLMD. Details on the combinations of parameters tested are provided in the Supporting Information. Training stops when the learning rate is less than 10^{-4} loss function unit.

As noted earlier, the intermediate cross sections observed in the AIMD at experimental conditions are used to benchmark the MLPES. For each candidate MLPES, hundreds of MLMD trajectories at the same initial conditions and trajectory propagation parameters (e.g., velocity Verlet integration, 0.5 fs time step) are carried out for direct comparison with AIMD. The model which reproduces the AIMD intermediate cross section within a 95% confidence interval[36] is used for the MLMD of interstellar conditions. More details on selecting MLPES for dynamics simulations are provided below.

The coordinates of the system in the trajectories were assigned with International Chemical Identifier (InChI) keys[40] on a frame-by-frame basis to categorize them into different intermediates. Similar to the previous AIMD study of CH + $H_2CCHCCH$,[3] InChI keys only characterize unique connectivity between atoms (constitutional isomers), so different conformers (e.g., rotational isomers) are ignored. Initiated from different geometries observed in AIMD trajectories corresponding to the same InChI key, geometry optimizations were attempted for each unique InChI key. The potential energy profile of the reaction was made from these optimal structures and used to assign the evolution of intermediates frame-by-frame for each trajectory, which is converted into a time series of InChI keys to characterize the reaction mechanism. Those transient, non-intermediate InChI keys (namely, frames corresponding to transient bond breakages) are counted towards the prior and following intermediates.

Table S1. Peak velocities (v_p) and speed ratios (S) of the methylidyne (CH; $X^2\Pi$), D1-methylidyne (CD; $X^2\Pi$), and indene (C_9H_8; X^1A') beams along with the corresponding collision energies (E_C) and center-of-mass angles (Θ_{CM})

Beam	v_p (m s^{-1})	S	E_C (kJ mol^{-1})	Θ_{CM} (degree)
C_9H_8	416 ± 15	10.2 ± 0.8		
CH	1897 ± 31	12.2 ± 0.7	22.1 ± 0.8	62.5 ± 0.7
CD	1882 ± 29	12.3 ± 0.8	23.2 ± 0.8	61.4 ± 0.7

Table S2. RRKM calculated product branching ratio (in %) for various initial complexes for the collision energies E_c of 0.5 and 22 kJ mol^{-1}.

		RRKM				
		Initial complex				
E_c (kJ mol^{-1})	Products	**i1**	**i2**	**i8**	**i9**	AI(ML)MD
0.5	**p1**	100	4.8	0	0	29.4
	**p2**	0	95.2	0	0	56.7
	**p3**	0	0	100	0	6.8
	**p4**	0	0	0	100	7.1
22	**p1**	100	4.7	0	0	29.2
	**p2**	0	95.3	0	0	45.7
	**p3**	0	0	100	0	8.9
	**p4**	0	0	0	100	16.2

Table S3. Parameter combinations (first three columns) of different MLPES and their errors in energy and force (fourth to fifth columns).

Nfeatures	Nwave	Cutoff (Å)	RMSE Energy (kJ/mol)	RMSE Force (kJ/mol/Å)
128	16	5	6.74	4.35
128	16	10	11.67	3.89
128	16	15	8.79	4.64
128	32	5	9.87	3.31
128	32	10	8.91	3.51
128	32	15	9.2	3.85
128	64	5	16.36	4.56
128	64	10	12.3	3.35

Table S4. The breakdown of the 169,131 geometries gathered from AIMD trajectories. Their energy and energy gradients are used in training the MLPES. Geometries are categorized by which intermediate they closely resemble or isomerize to/from. Geometries looking like reactants (e.g., CH) or products (e.g., CH_2, H), even just transiently, are denoted "vX" where X is the separated species.

Closest Species	Number of Geometries
**i1.x**	15797
**i2.x**	3980
**i3**	35685
**i5**	44275
**i8.x**	6272
**i9.x**	18845
**iC**	3148
**vCH_2**	7388
**vCH**	28986
**vH**	4755

Table S5. Relative intermediate populations (in %) at the start (t=0) and end (t=500 fs). Isomers of similar connectivity are under the same label (e.g., **i1** includes all **i1.x**). All intermediates other than **i1.x**, **i2.x**, **i.3**, **i5**, **i8.x**, and **i9.x** are grouped into **iO**.

	Collision Energy	i3	iO	i5	i9	i8	i1	i2
**AIMD (t=0)**	22 kJ mol^{-1}	0.0	5.4	0.0	18.0	11.4	23.6	41.6
**MLMD (t=0)**	22 kJ mol^{-1}	0.0	2.7	0.0	15.4	8.6	26.7	46.6
**MLMD (t=0)**	0.5 kJ mol^{-1}	0.0	0.0	0.0	7.1	6.8	26.5	59.5
**AIMD (t=0.5ps)**	22 kJ mol^{-1}	39.7	5.6	4.9	16.8	11.4	19.7	1.9
**MLMD (t=0.5ps)**	22 kJ mol^{-1}	45.2	2.9	10.1	15.7	8.6	16.0	1.4
**MLMD (t=0.5ps)**	0.5 kJ mol^{-1}	58.7	0.1	10.1	7.1	6.8	16.4	0.8

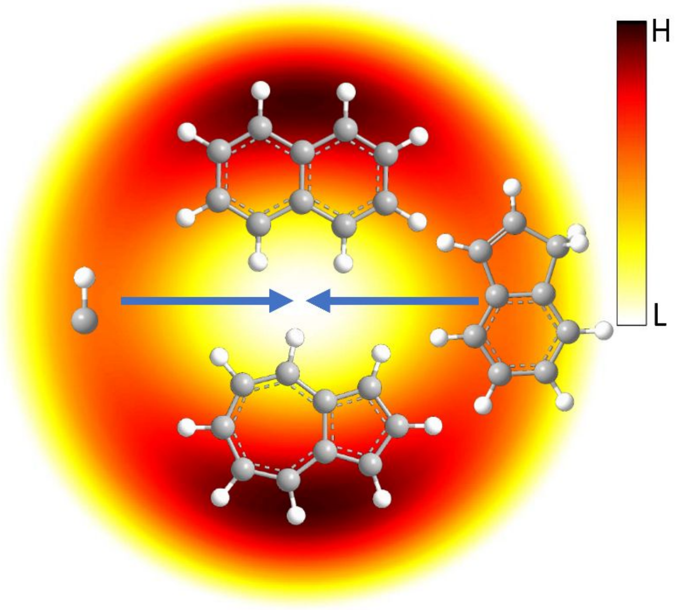

Figure S1. The flux contour map in the CH – C_9H_8 reaction which leads to the formation of naphthalene and azulene.

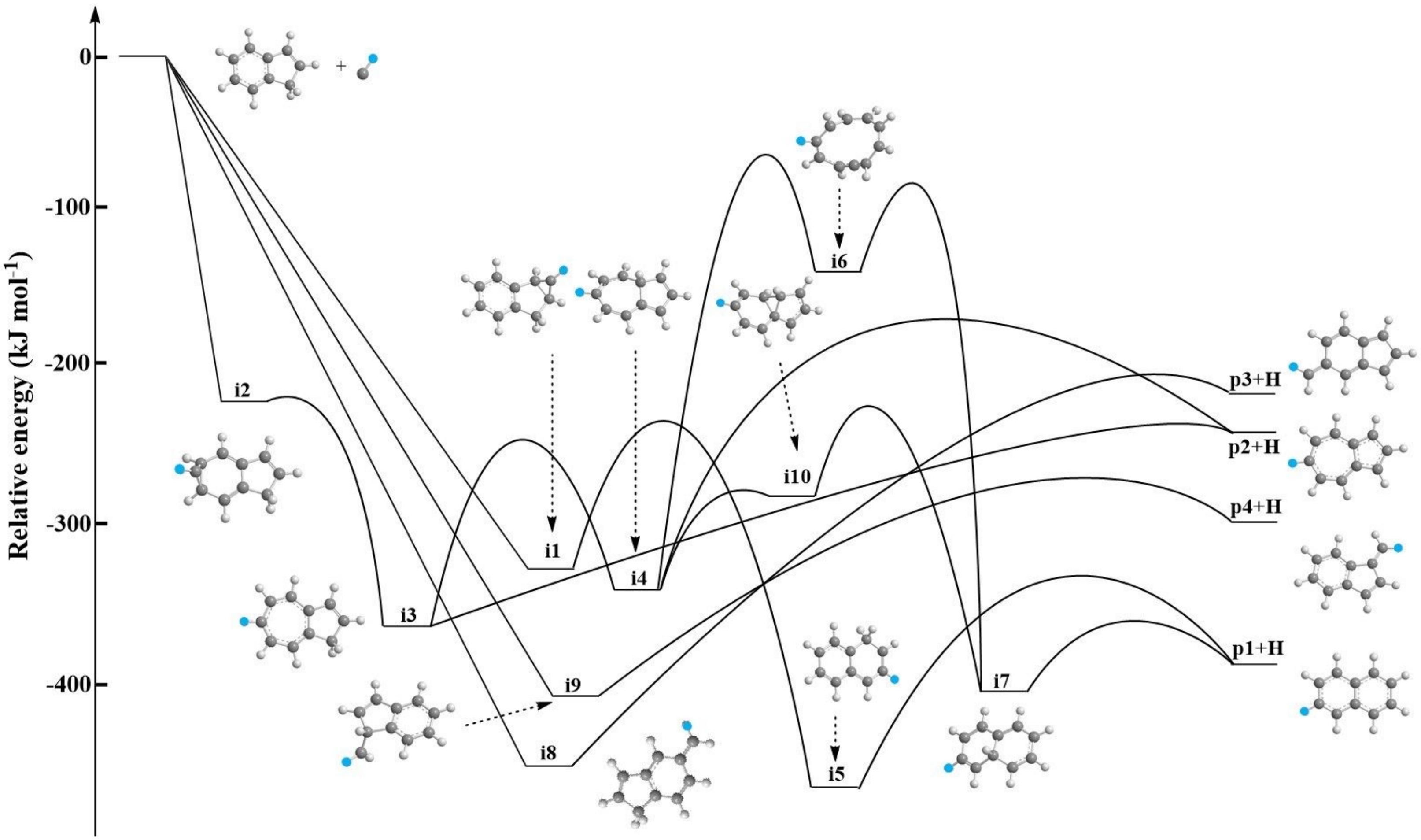

Figure S2. Potential energy surface (PES) for the reaction of D1-methylidyne (CD) with indene (C_9H_8).

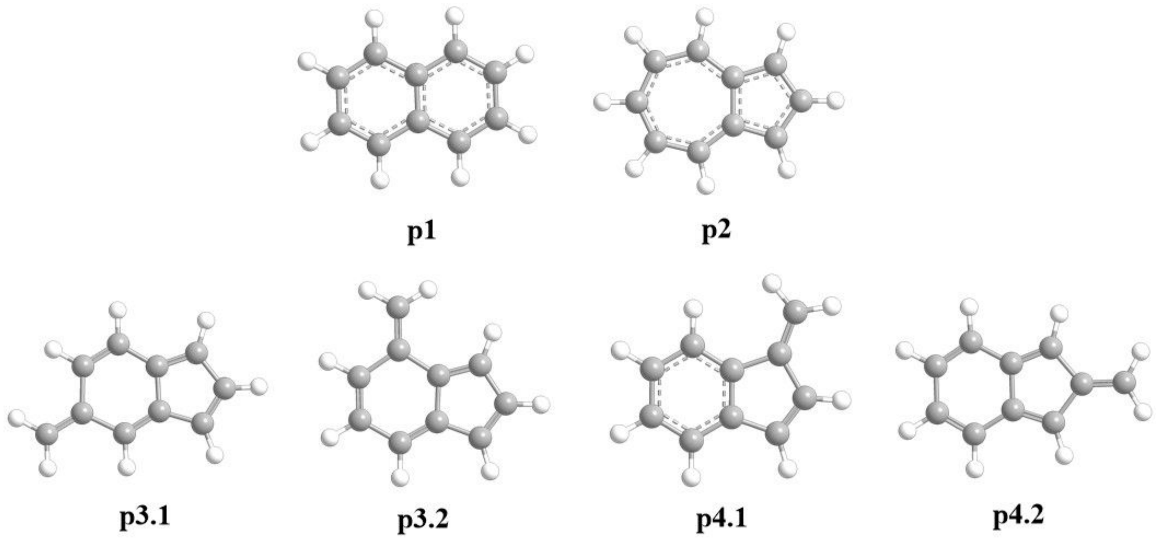

Figure S3. A complete list of products in the CH – C_9H_8 reaction.

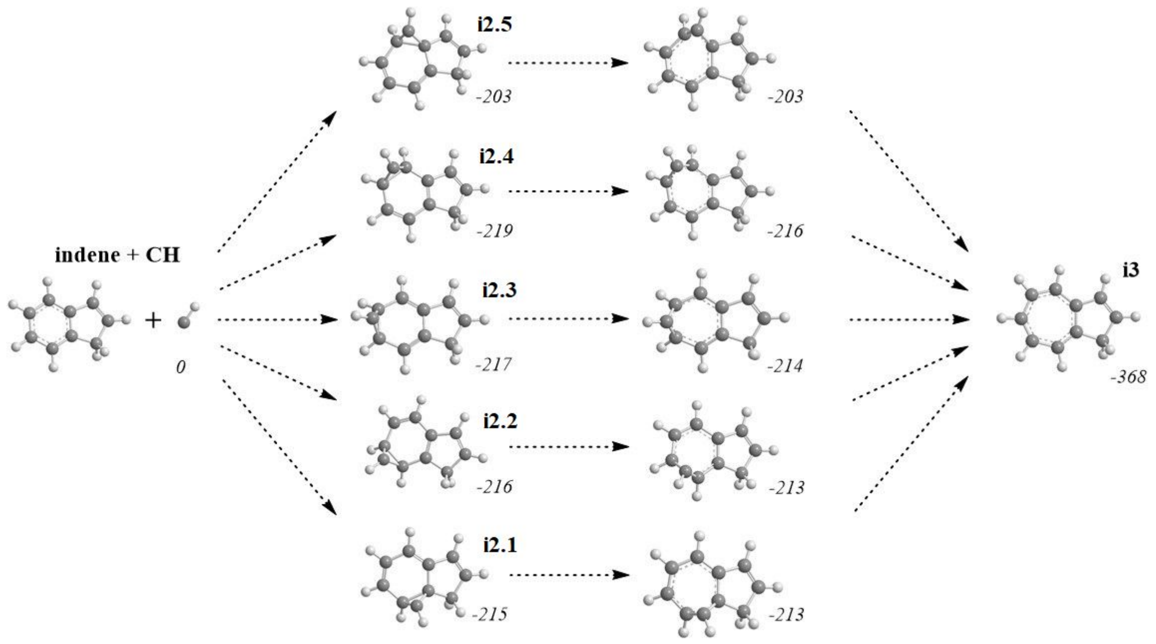

Figure S4. The pathways via the addition of the CH radical to different C-C bonds in the six-membered ring of indene lead to **i3**.

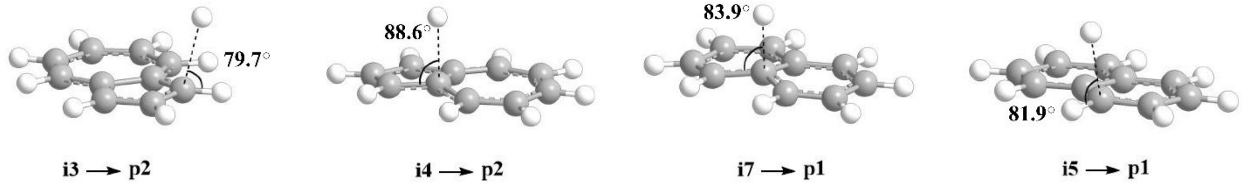

Figure S5. Computed geometries of the exit transtition states forming azulene (**p2**) and naphthalene (**p1**). Angles of the departing hydrogen atoms are given in degrees with respect to the rotation plane of the decomposing complex.

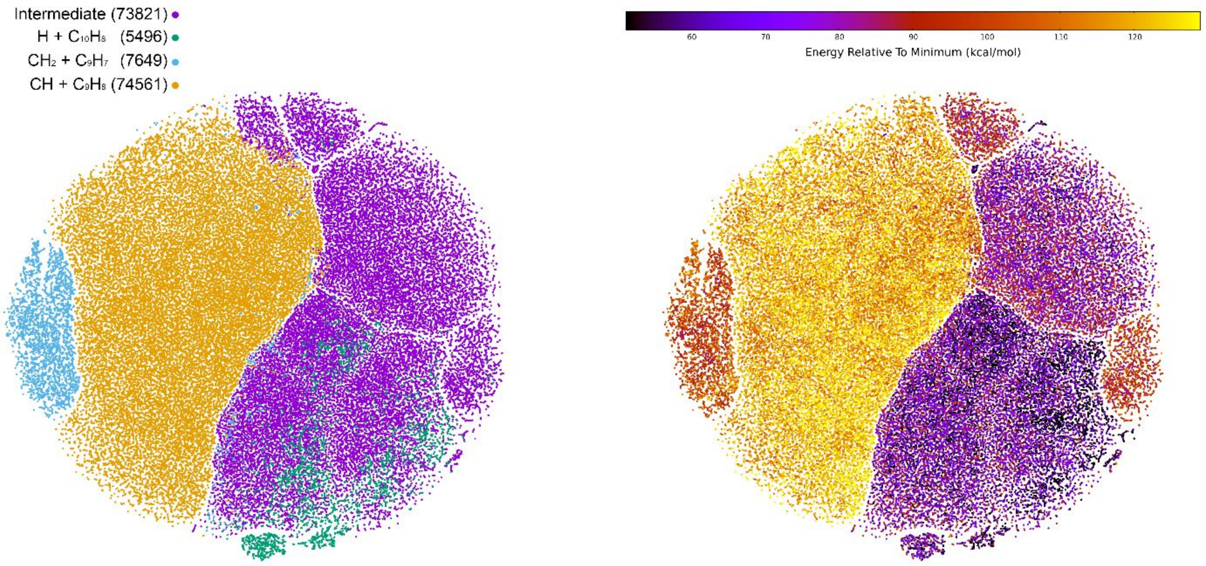

Figure S6. A T-SNE clustering of the training set, with each point representing a configuration and distances between points defined with the sorted DMD. On the left, points are colored by molecular fragments—orange, blue, green, and purple are reactant-like, CH_2 loss, H loss, and intermediate-like, respectively—with populations indicated in parentheses. On the right, the same points are colored by relative energy (with respect to the global minimum).

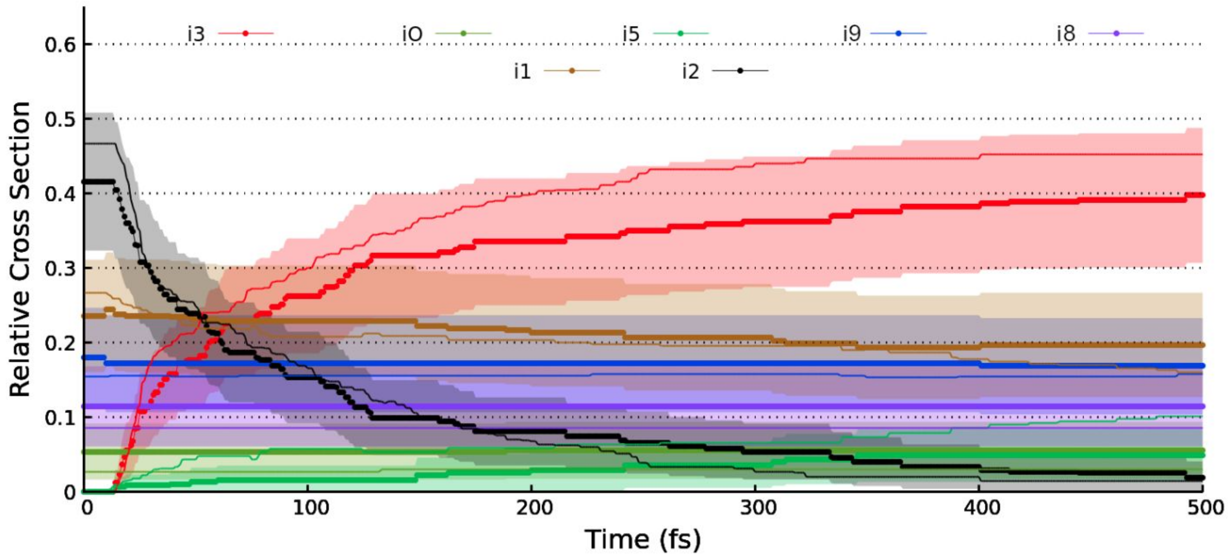

Figure S7. Relative cross sectional areas for the AIMD (thick line) and MLMD (thin line) at experimental condition. Filled curves around the AIMD result indicate one standard deviation. Isomers of similar connectivity are under the same label (e.g., **i1** includes all **i1.x**). All intermediates other than **i1.x**, **i2.x**, **i.3**, **i5**, **i8.x**, and **i9.x** are grouped into **iO**.

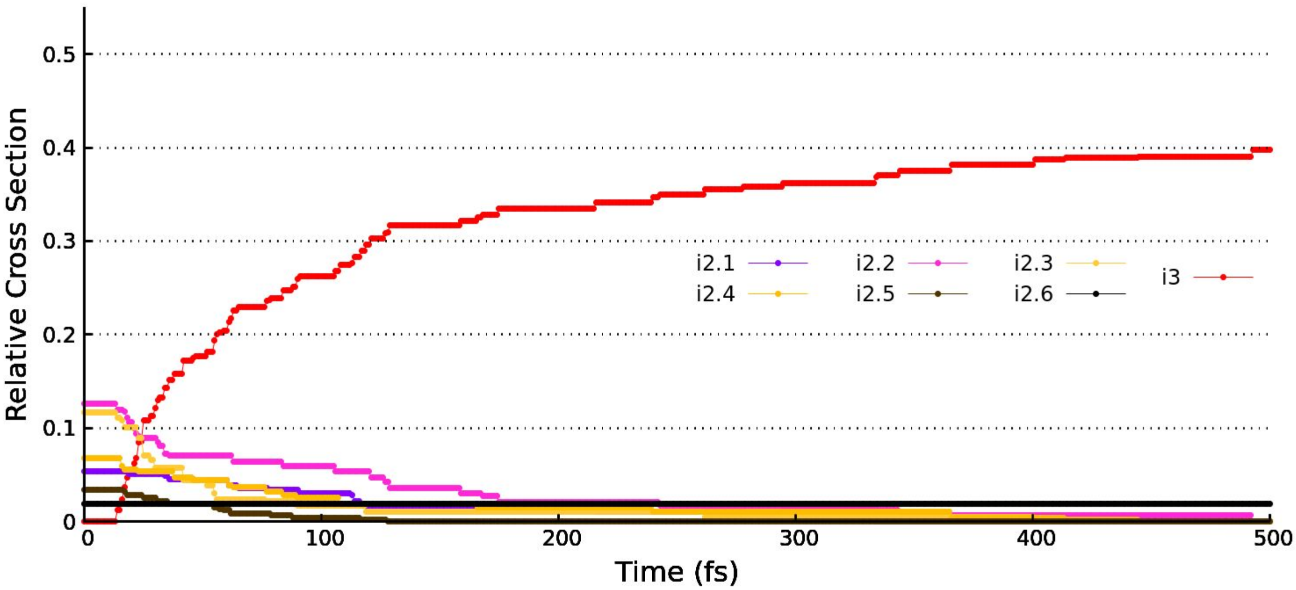

Figure S8. An example of the detailed breakdown of the relative cross section by isomers (**i2.x**), which nearly all isomerize to **i3**.

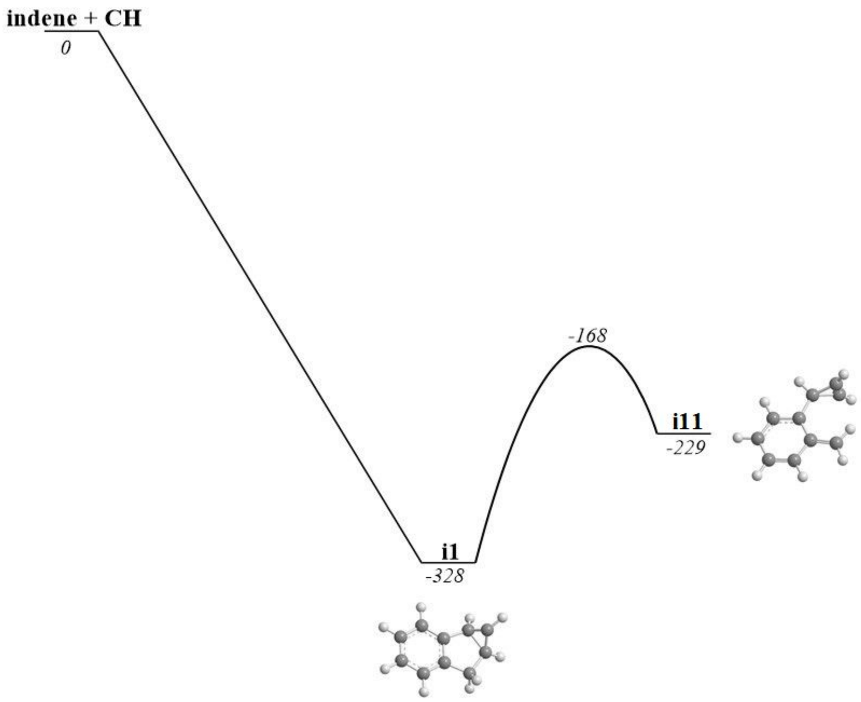

Figure S9. The formation of the intermediate **i11** via the C-C bond rupture of the five-membered ring of **i1.3**.

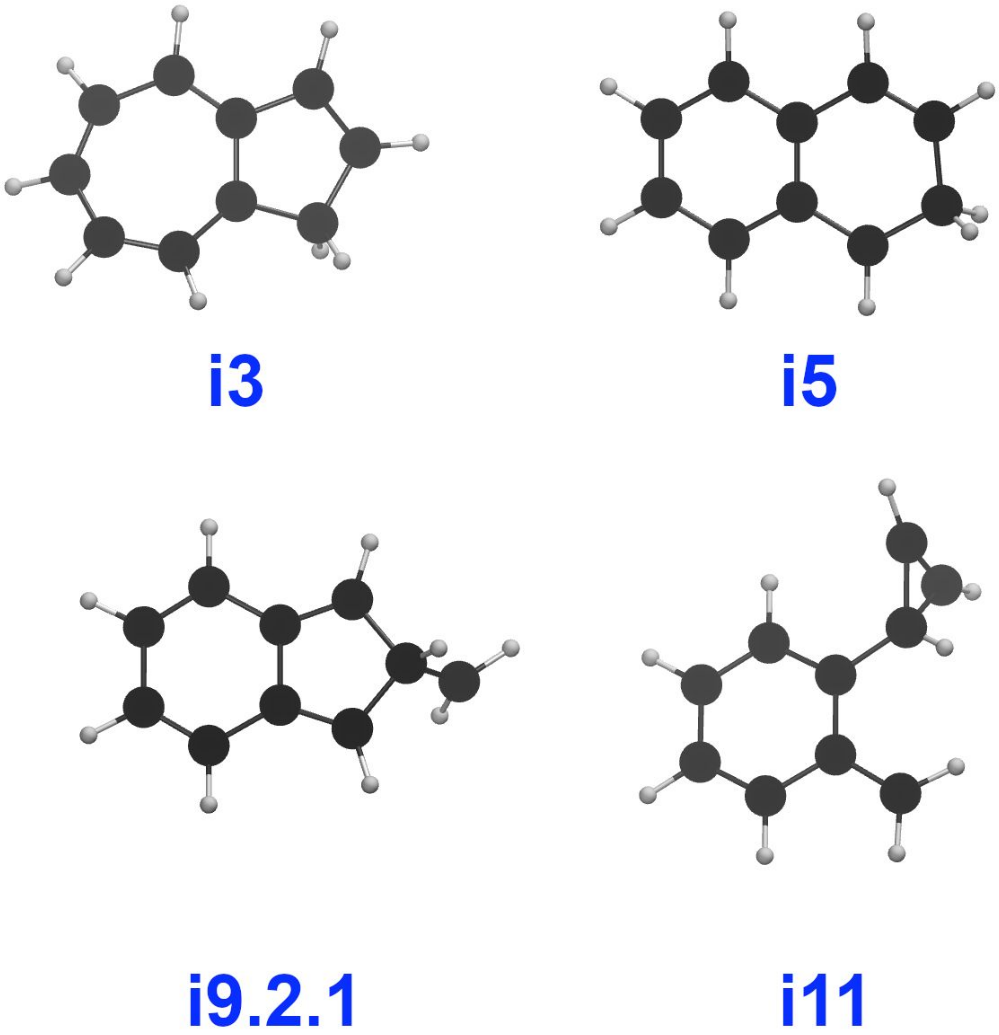

Figure S10. Other intermediates that are not directly formed after the collision.

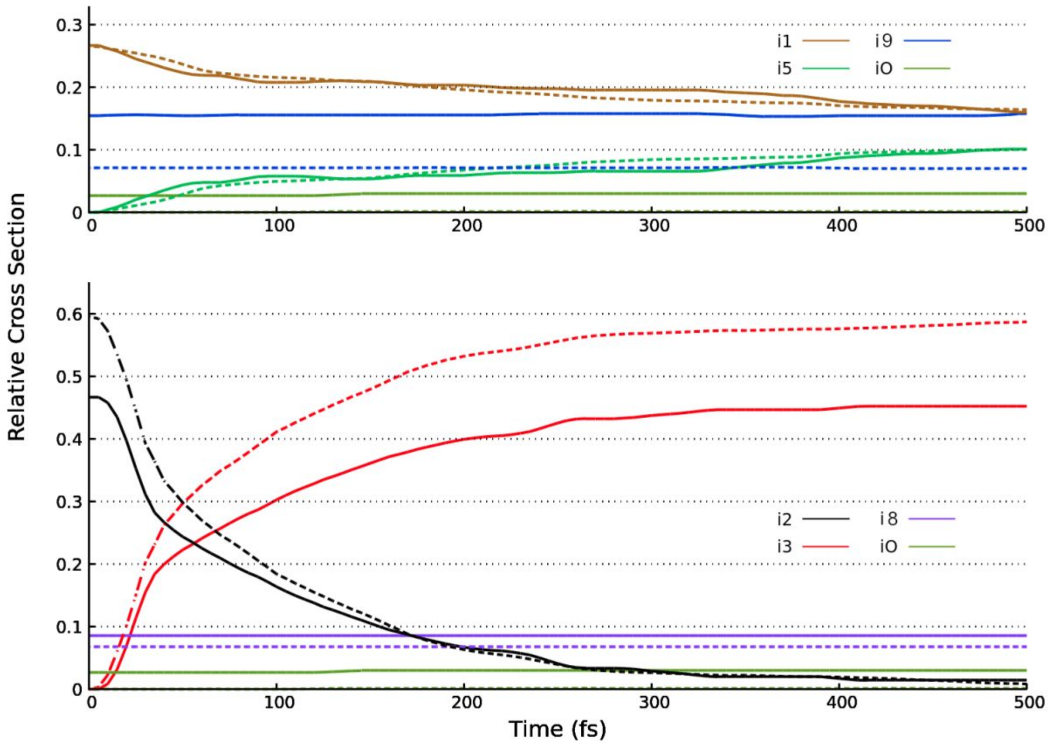

Figure S11. Relative cross sectional areas for the machine-learned molecular dynamics (MLMD) simulations at collision energies of 0.5 kJ mol^{-1} (dashed line) and 22 kJ mol^{-1} (solid line) for intermediates related to the five-membered ring (above) and the six-membered ring (below). Isomers of similar connectivity are under the same label (e.g., **i1** includes all **i1.x**).

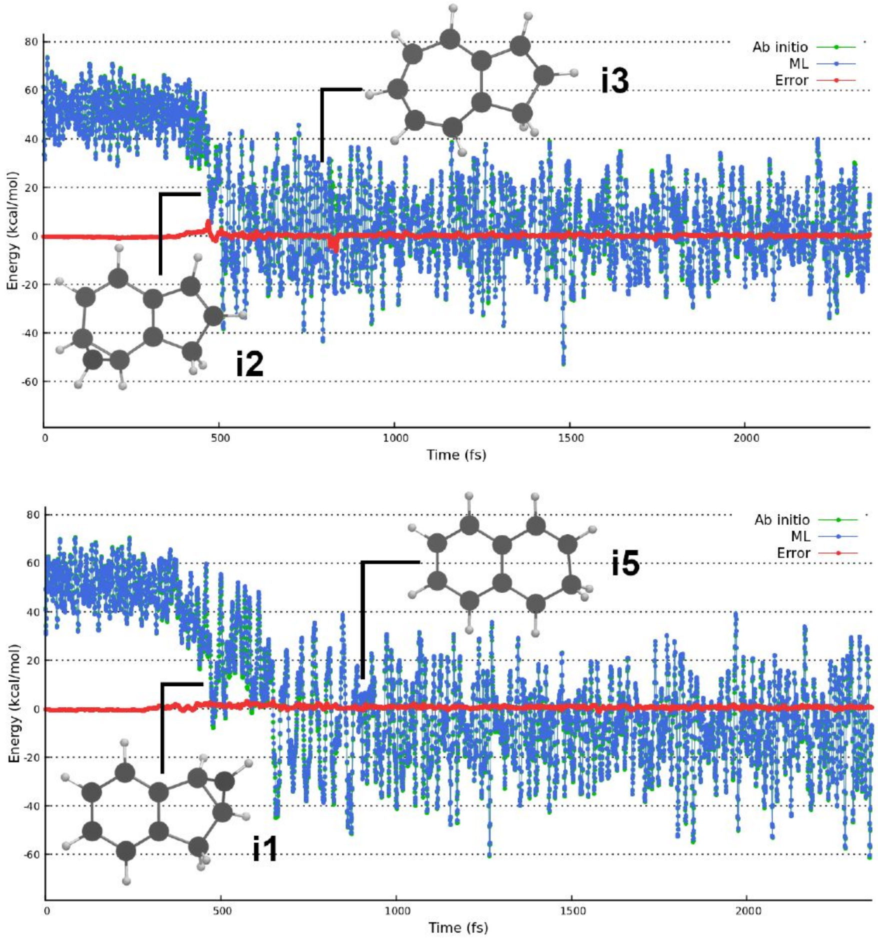

Figure S12. Comparison of *ab initio* (green) and ML predicted (blue) energies over example AIMD trajectories for (left) **r** to **i2** to **i3** formation and (right) **r** to **i1** to **i5** formation. The difference in energy prediction or "error" is overlaid to scale in red.

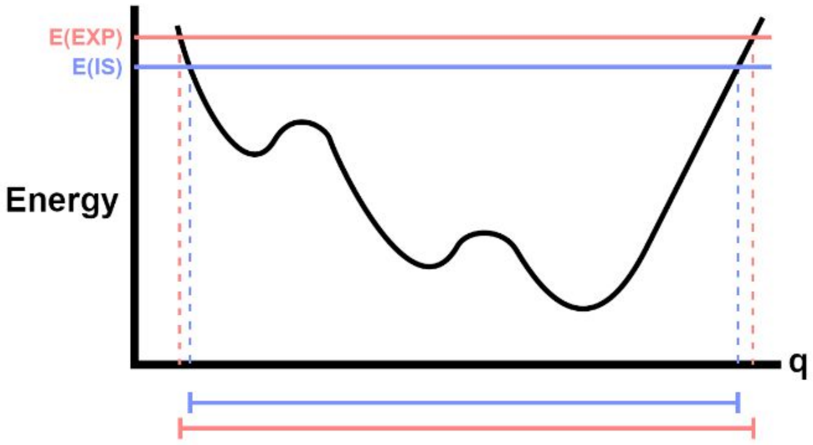

Figure S13. A pedagogical diagram (not to scale) of the geometrical space under study. The experimental (EXP) and interstellar (IS) conditions correspond to two different amounts of total energy accessible to the system, the horizontal lines E(EXP) in red and E(IS) in blue. These intersect the underlying ML potential (black curve), demarcating the geometrical space each corresponds to, shown as ranges on the x axis.

Table S6. Optimized Cartesian coordinates and vibrational frequencies for all intermediates, transition states, reactants and products involved in the reactions of the methylidyne radical (CH) with indene (C_9H_8).

Reactants:

Indene

Coordinates:

C	0.428451	-0.617267	0.000000
C	0.000000	0.725770	0.000000
C	-0.501155	-1.655555	0.000000
C	1.893788	-0.628891	0.000000
C	2.358314	0.632232	0.000000
C	1.216877	1.620714	0.000000
C	-1.860037	-1.338688	0.000000
C	-2.282522	-0.008741	0.000000
C	-1.350923	1.034599	0.000000
H	-0.178413	-2.691147	0.000000
H	2.496275	-1.528580	0.000000
H	3.399177	0.927692	0.000000
H	1.246867	2.278669	0.877872
H	1.246867	2.278669	-0.877872
H	-2.596627	-2.134465	0.000000
H	-3.342726	0.218012	0.000000
H	-1.688191	2.066115	0.000000

Frequencies:

193.7537	211.1513	388.0297
396.5234	427.1579	542.6231
563.2046	605.1407	707.3362
734.0126	744.9778	784.0228
844.8027	871.0683	873.5357
934.9869	954.1984	956.1294
965.0813	988.2425	1042.9181
1089.0905	1132.8889	1148.6344
1177.5121	1186.0450	1227.7377
1249.4029	1314.9832	1345.4963
1391.2775	1437.2311	1489.2461
1492.6995	1603.5538	1637.3160
1652.2007	3014.1781	3035.9821
3156.3092	3162.8467	3173.7951
3186.5582	3189.5704	3213.3063

T1 diagnostic:

0.01063804

CH

Coordinates:

C	0.000000	0.000000	0.161147
H	0.000000	0.000000	-0.966884

Frequencies:

2803.8393

T1 diagnostic:

0.00849331

Intermediates:

i1

Coordinates:

C	-0.008366	-0.619343	-0.159136
C	0.119982	0.770775	-0.040598
C	1.115151	-1.438919	-0.134247
C	-1.445161	-1.022121	-0.286774
C	-2.244674	0.320046	-0.251160
C	-1.222556	1.463340	-0.159301
C	2.375562	-0.857829	0.018524
C	2.504447	0.523900	0.154288
C	1.374631	1.344776	0.128115
H	1.015982	-2.515326	-0.225900
H	-1.705339	-1.853198	-0.936958
H	-3.116466	0.475257	-0.880806
H	-1.262019	2.082555	-1.062377
H	-1.429180	2.119606	0.692078
H	3.259256	-1.486091	0.036825
H	3.487898	0.964580	0.274265
H	1.481187	2.420627	0.223807
C	-2.326899	-0.656179	0.836916
H	-3.184016	-1.178683	1.239313

Frequencies:

137.9465	177.6177	311.4775
360.0452	370.3263	439.9260
515.0265	543.8210	589.2180
658.7600	689.0521	733.4996
736.8234	773.1455	777.3932
820.7732	863.1570	876.0169
944.2939	961.2734	985.5485
988.4437	1026.0177	1046.1026

1059.2354	1077.0476	1120.6699
1174.4974	1178.1170	1179.8733
1197.1898	1229.7938	1254.9576
1274.9784	1332.5118	1339.7720
1390.7898	1479.0640	1490.3905
1509.2136	1624.6527	1645.3877
3022.5740	3056.0821	3122.7889
3128.4070	3154.8367	3161.4785
3172.2979	3184.9848	3198.8505

T1 diagnostic:

0.01158094

i2

Coordinates:

C	-0.488076	-0.751463	-0.010618
C	-0.507399	0.712350	-0.014537
C	0.643628	-1.460923	-0.213053
C	-1.881710	-1.195967	0.056629
C	-2.711138	-0.142613	0.112831
C	-1.953842	1.166466	0.082023
C	1.964432	-0.808955	-0.236355
C	1.942408	0.818779	-0.264063
C	0.601422	1.436863	-0.239500
H	0.606935	-2.526386	-0.417512
H	-2.181666	-2.236686	0.060259
H	-3.791000	-0.195155	0.171617
H	-2.252100	1.792849	-0.767502
H	-2.138948	1.760929	0.985770
H	2.724784	-1.316071	-0.829670
H	2.681933	1.329610	-0.879966
H	0.545614	2.502520	-0.443203
C	2.453078	0.031606	0.858848
H	3.427631	0.051537	1.326974

Frequencies:

141.2551	164.1203	245.4998
320.1024	349.6305	385.4951
453.7743	487.8391	538.9385
565.3383	677.9220	688.5550
725.2519	754.2051	757.0436
822.9068	829.4659	837.0425
853.0505	953.4898	963.8165
974.1978	981.3595	1025.1209
1029.1159	1057.2975	1091.2238
1121.0920	1153.9057	1164.7619

1195.7108	1217.4550	1267.6432
1312.5424	1337.9993	1366.2470
1393.0452	1405.1357	1461.5915
1626.5488	1640.6654	1712.4757
3007.9741	3028.8293	3083.7767
3088.7361	3147.0640	3156.9496
3183.9395	3195.8116	3206.7225

T1 diagnostic:

0.01234924

i3

Coordinates:

C	-0.408644	-1.667068	0.000000
C	1.360060	0.969067	0.000000
C	2.326743	-0.108245	0.000000
C	2.114396	-1.449294	0.000000
C	0.864021	-2.175076	0.000000
C	-0.833695	-0.315095	0.000000
C	-2.208506	0.113863	0.000000
C	-2.306173	1.465846	0.000000
C	-0.928776	2.074986	0.000000
C	0.000000	0.867382	0.000000
H	-1.212899	-2.400195	0.000000
H	1.779182	1.971441	0.000000
H	3.364824	0.212219	0.000000
H	3.003269	-2.073959	0.000000
H	0.954639	-3.256258	0.000000
H	-3.044919	-0.575272	0.000000
H	-3.220382	2.042785	0.000000
H	-0.750138	2.710521	0.877227
H	-0.750138	2.710521	-0.877227

Frequencies:

114.0849	167.0548	233.2150
331.0939	362.8598	404.3222
425.8533	492.7408	560.0381
570.3201	633.5500	665.5196
675.7607	740.9175	758.4319
801.6831	858.4976	891.0132
922.9168	929.8398	947.7750
950.6488	977.5241	980.7536
994.9989	1048.4297	1081.2286
1129.1956	1152.7526	1204.4254
1242.8173	1261.6149	1293.2192
1346.3617	1383.2985	1433.3511

1449.0519	1467.7706	1512.8195
1557.6412	1607.8466	1653.8999
3001.8837	3019.9205	3126.0487
3130.7110	3142.7906	3156.8410
3169.0304	3183.0962	3216.8487

T1 diagnostic:

0.01232503

i4

Coordinates:

C	0.643679	1.598462	0.004503
C	0.460897	-1.552134	-0.238074
C	1.785074	-1.300371	-0.331393
C	2.484843	-0.107909	0.060886
C	1.974775	1.169830	0.186334
C	-0.495869	0.792663	0.004447
C	-1.799818	1.158571	-0.321965
C	-2.678199	0.039248	-0.141228
C	-1.975132	-1.035007	0.305076
C	-0.524885	-0.659633	0.475969
H	0.490105	2.662679	-0.154289
H	0.074034	-2.473681	-0.661397
H	2.404743	-2.080095	-0.768246
H	3.563817	-0.203060	0.141311
H	2.706124	1.958211	0.343534
H	-2.101346	2.138425	-0.669847
H	-3.742223	0.057183	-0.340778
H	-2.369059	-2.017202	0.530104
H	-0.278389	-0.664775	1.552280

Frequencies:

111.9535	150.2404	293.4875
312.5465	378.2583	382.5006
495.8724	550.4266	574.1632
606.5408	683.7014	703.6213
715.1460	775.2725	801.7516
818.2162	853.4098	870.1643
907.3149	937.9944	952.6046
959.5848	979.9815	1004.7802
1032.5688	1050.7679	1106.5117
1129.6744	1205.3534	1214.7491
1235.0776	1248.6241	1262.9761
1337.3354	1349.8193	1388.6717
1420.5943	1429.8434	1489.6357
1541.4682	1572.9135	1635.8832

2932.1533	3125.6296	3131.5490
3144.5598	3156.6728	3162.7909
3188.5952	3200.8768	3216.2306

T1 diagnostic:

0.01320732

i5

Coordinates:

C	-1.367679	-1.324149	0.000000
C	1.351720	1.378016	0.000000
C	-1.170946	1.446288	0.000000
C	-2.424848	0.839190	0.000000
C	-2.519274	-0.555296	0.000000
C	-0.089006	-0.725145	0.000000
C	1.106951	-1.516899	0.000000
C	2.374959	-0.912706	0.000000
C	2.525747	0.447863	0.000000
C	0.000000	0.691391	0.000000
H	-1.436181	-2.407220	0.000000
H	1.417358	2.052895	0.868000
H	-1.101649	2.530268	0.000000
H	-3.322196	1.447390	0.000000
H	-3.491931	-1.034560	0.000000
H	1.017884	-2.597076	0.000000
H	3.257165	-1.545053	0.000000
H	3.516447	0.889134	0.000000
H	1.417358	2.052895	-0.868000

Frequencies:

82.9080	171.4444	250.1674
357.6328	429.3457	471.4283
485.4929	505.9855	541.4510
612.7023	660.7264	713.8472
747.6068	751.5604	791.8220
794.5493	871.0539	920.8973
931.6614	954.3490	959.3817
966.7203	984.7799	1056.8353
1092.1699	1139.1744	1175.4258
1179.0778	1200.9442	1202.3256
1233.8800	1277.2436	1309.3047
1355.7613	1393.4245	1438.8648
1445.1476	1473.1892	1515.8089
1559.4845	1599.1565	1629.6533
2955.4603	2955.5539	3148.7398
3152.4502	3157.9410	3171.1422

3172.3042	3182.6696	3186.8482

T1 diagnostic:

0.01373922

i6

Coordinates:

C	0.825099	1.666877	-0.393564
C	0.097256	-1.556353	0.477394
C	1.368168	-1.230634	0.751009
C	2.283678	-0.380447	-0.014481
C	2.053184	0.862822	-0.476003
C	-0.256793	1.450603	0.312723
C	-0.701713	-1.348070	-0.746993
C	-1.898176	-0.681630	-0.801136
C	-2.350338	0.275359	0.145601
C	-1.451744	1.167817	0.821961
H	0.817547	2.548750	-1.037248
H	-0.436488	-2.105599	1.253676
H	1.786673	-1.625172	1.676592
H	3.293554	-0.767391	-0.139024
H	2.868953	1.354558	-0.998509
H	-0.392720	-1.917446	-1.620936
H	-2.559344	-0.904555	-1.637815
H	-3.416920	0.414903	0.297511
H	-1.772980	1.643898	1.746687

Frequencies:

111.3418	146.9818	176.4865
190.7773	256.2236	264.6312
339.2007	379.6083	426.2613
494.2404	575.6067	601.9052
671.5432	689.1488	757.4047
775.3327	816.7815	820.5470
845.1425	875.3646	885.6294
904.2447	945.2662	963.4546
980.8918	997.8951	1034.7321
1098.4151	1147.8108	1208.7930
1259.0058	1269.5383	1311.2898
1372.0870	1400.3737	1417.6279
1445.5217	1459.9689	1517.5366
1660.4208	1684.3458	1924.9929
3073.7385	3088.4493	3104.7940
3109.5217	3115.7873	3119.1528
3131.1092	3149.4422	3150.6690

T1 diagnostic:

0.01664192

i7

Coordinates:

C	0.230153	1.408675	1.237401
C	-0.545895	-0.638764	0.000000
C	-0.246721	-1.389141	1.271999
C	0.065775	-0.730488	2.409080
C	0.230153	0.697347	2.419457
C	-0.000244	0.779715	0.000000
C	-0.246721	-1.389141	-1.271999
C	0.065775	-0.730488	-2.409080
C	0.230153	0.697347	-2.419457
C	0.230153	1.408675	-1.237401
H	0.480717	2.465482	1.243047
H	-1.660213	-0.512137	0.000000
H	-0.356903	-2.468587	1.259927
H	0.225693	-1.281844	3.329773
H	0.442861	1.199813	3.355667
H	-0.356903	-2.468587	-1.259927
H	0.225693	-1.281844	-3.329773
H	0.442861	1.199813	-3.355667
H	0.480717	2.465482	-1.243047

Frequencies:

116.0481	152.8987	326.5242
351.0553	441.8448	468.9892
470.4058	514.7118	565.6079
609.2132	665.8677	679.7140
707.6432	770.7992	780.8770
793.8643	869.8404	919.2411
928.0372	954.0079	971.8657
974.8751	996.6913	998.9462
1070.2659	1073.8429	1109.0429
1158.9670	1174.4314	1182.1650
1205.0267	1258.7304	1262.4462
1334.0379	1340.6515	1400.6684
1417.3456	1468.6078	1507.4989
1515.1783	1631.8398	1633.1531
2695.1295	3147.8322	3151.3849
3153.3226	3155.5384	3171.1655
3172.8844	3186.9783	3188.0122

T1 diagnostic:

0.01352574

i10

Coordinates:

C	0.853770	1.419441	0.207874
C	0.131654	-0.970207	-0.532395
C	1.551462	-1.261436	-0.507350
C	2.465086	-0.405772	0.016856
C	2.113729	0.952331	0.359749
C	-0.278113	0.542797	-0.094217
C	-1.563244	1.013750	-0.650010
C	-2.599381	0.206997	-0.184522
C	-2.105675	-0.770388	0.673931
C	-0.636756	-0.657878	0.759509
H	0.647069	2.481130	0.293397
H	-0.466683	-1.421924	-1.316836
H	1.881238	-2.180168	-0.982819
H	3.511699	-0.689090	0.042773
H	2.910288	1.637464	0.628477
H	-1.653435	1.837524	-1.345343
H	-3.637846	0.308429	-0.473535
H	-2.685002	-1.533085	1.176308
H	-0.102514	-0.858088	1.681035

Frequencies:

171.8436	213.1531	235.5657
298.2880	392.3555	443.2464
521.2358	531.9446	567.1336
628.0164	659.8257	683.6904
702.0956	759.0581	771.7496
801.6188	828.9710	894.2016
931.4257	933.5670	953.5577
973.7625	981.3131	987.0589
1025.0316	1046.8409	1086.2091
1089.4862	1092.3988	1173.1613
1196.7054	1204.8584	1238.9415
1284.8186	1340.1158	1346.4992
1397.0946	1423.5123	1463.2320
1476.5507	1560.8153	1661.7706
3140.9778	3151.8091	3157.3120
3159.6044	3170.0239	3182.2409
3191.1430	3207.0385	3218.5810

T1 diagnostic:

0.01210221

Products:

p1 (naphthalene)

Coordinates:

C	0.000000	0.000000	0.715661
C	0.000000	0.000000	-0.715661
C	0.000000	1.243465	1.400275
C	0.000000	-1.243465	1.400275
C	0.000000	1.243465	-1.400275
C	0.000000	-1.243465	-1.400275
C	0.000000	2.429373	0.707395
C	0.000000	-2.429373	0.707395
C	0.000000	2.429373	-0.707395
C	0.000000	-2.429373	-0.707395
H	0.000000	1.241873	2.485452
H	0.000000	-1.241873	2.485452
H	0.000000	1.241873	-2.485452
H	0.000000	-1.241873	-2.485452
H	0.000000	3.372182	1.242827
H	0.000000	-3.372182	1.242827
H	0.000000	3.372182	-1.242827
H	0.000000	-3.372182	-1.242827

Frequencies:

173.5767	186.4701	365.7431
395.8409	479.6401	488.2899
518.9866	519.9284	634.9454
636.1375	729.2844	773.2753
786.9170	798.5095	808.8427
849.6195	896.9289	950.9856
957.0662	974.3852	992.5896
999.9182	1035.6100	1046.3292
1151.0802	1169.1655	1171.8897
1185.2344	1232.3707	1270.0589
1287.0697	1391.2459	1398.5774
1418.0335	1490.5762	1491.7005
1548.6462	1613.8797	1641.3706
1671.2250	3155.9872	3157.7601
3160.0754	3163.5291	3174.0822
3175.4298	3186.9388	3188.1020

T1 diagnostic:

0.01071909

p2 (azulene)

Coordinates:

C	0.000000	0.749633	0.553208
C	0.000000	-0.749633	0.553208
C	0.000000	1.148409	1.899320
C	0.000000	-1.148409	1.899320
C	0.000000	1.592432	-0.551625
C	0.000000	-1.592432	-0.551625
C	0.000000	1.263804	-1.907998
C	0.000000	-1.263804	-1.907998
C	0.000000	0.000000	2.704613
C	0.000000	0.000000	-2.499250
H	0.000000	0.000000	3.787071
H	0.000000	0.000000	-3.586206
H	0.000000	2.172524	2.245658
H	0.000000	-2.172524	2.245658
H	0.000000	2.656321	-0.324086
H	0.000000	-2.656321	-0.324086
H	0.000000	2.103905	-2.595518
H	0.000000	-2.103905	-2.595518

Frequencies:

162.9250	170.3153	320.0281
338.1632	411.6873	429.1417
498.2979	574.5125	611.7938
679.2767	724.3187	740.7240
744.1370	782.2037	795.5349
832.8631	875.3884	915.2607
939.9450	961.2449	974.9983
992.7215	1008.4363	1022.5941
1064.0122	1080.9901	1185.8101
1238.2533	1242.9690	1300.5683
1321.8208	1335.4787	1420.0467
1427.9204	1486.8531	1488.8581
1532.3461	1579.8587	1632.7469
1643.5183	3129.3798	3131.5186
3139.0378	3158.8245	3167.5631
3195.5713	3213.0415	3222.2338

T1 diagnostic:

0.01253578

Barriers:

ts1

Coordinates:

C	0.485573	0.745377	0.010664
C	0.503239	-0.706812	0.007577

C	-0.643394	1.462265	-0.230359
C	1.880678	1.193193	0.056199
C	2.712104	0.141291	0.106561
C	1.949287	-1.163498	0.086117
C	-1.966787	0.860275	-0.209134
C	-1.944725	-0.869390	-0.242002
C	-0.602002	-1.437525	-0.259099
C	-2.439747	-0.031548	0.838908
H	-0.579556	2.505846	-0.523353
H	2.177794	2.234750	0.052486
H	3.792366	0.192099	0.155619
H	2.234090	-1.792363	-0.766223
H	2.142803	-1.757990	0.988210
H	-2.733935	1.376967	-0.785486
H	-2.691107	-1.388224	-0.842931
H	-0.518625	-2.481726	-0.548026
H	-3.429185	-0.051134	1.277107

Frequencies:

-317.9648	149.1276	166.1295
285.6539	339.4221	379.9001
441.8241	456.9488	527.8476
562.0892	687.4577	692.8584
727.8244	747.6674	757.4340
819.8119	827.8426	853.7655
863.0561	952.5009	960.2474
963.4494	969.1672	1022.7604
1030.2059	1046.4132	1113.0173
1152.0471	1158.1157	1168.8207
1199.8122	1222.2119	1267.9609
1314.8128	1344.3795	1365.4463
1398.5113	1425.4913	1458.0421
1604.2267	1628.9893	1691.7775
3008.5248	3030.3333	3087.5345
3090.7583	3143.6766	3153.1974
3183.6661	3184.3807	3207.6965

T1 diagnostic:

-385.35400025

ts2

Coordinates:

C	2.735004	0.055174	-0.077807
C	0.525636	-0.697201	0.078678

C	0.533936	0.744712	0.003409
C	-2.541010	-0.005838	-0.008367
C	1.954355	-1.108707	0.027282
C	-0.595633	-1.614550	-0.045459
C	-1.923798	-1.274789	-0.074497
C	1.873387	1.157613	-0.090217
C	-0.606743	1.617900	0.032099
C	-1.937522	1.269763	0.038248
H	3.814411	0.082308	-0.087421
H	-3.625636	-0.012756	-0.022041
H	2.271946	-2.140300	-0.028680
H	-0.341976	-2.667829	-0.111538
H	-2.611447	-2.111812	-0.168554
H	2.187673	2.193238	-0.113905
H	-0.376452	2.679139	0.029301
H	-2.635699	2.102680	0.060535
H	1.211519	-0.989127	1.142081

Frequencies:

-1222.6590	120.5263	187.8815
310.6289	334.9586	390.9415
419.4132	497.8002	573.1279
595.9050	657.3507	672.9654
706.6095	753.7687	764.8242
771.6775	818.3078	863.0521
895.1530	912.4457	915.9767
933.2029	974.4746	976.7869
1038.8309	1077.6224	1108.3513
1135.4702	1188.9703	1211.6793
1241.1515	1247.4733	1274.1636
1342.4385	1383.9729	1412.5767
1455.0573	1468.4665	1473.2764
1529.2210	1573.1770	1602.1441
2014.7783	3129.8864	3133.5556
3153.9582	3158.0674	3170.1477
3197.6494	3217.8394	3235.5394

T1 diagnostic:

0.01302568

ts3

Coordinates:

C	0.627184	1.597578	0.039544
C	0.539823	-1.581996	-0.072248
C	1.905132	-1.292727	-0.042965
C	2.531251	-0.046877	0.015198

C	1.974702	1.231633	0.053812
C	-0.497748	0.784525	-0.014033
C	-1.835621	1.224199	-0.037830
C	-2.673036	0.108667	-0.089203
C	-1.900863	-1.071977	-0.079898
C	-0.537926	-0.707193	-0.058471
H	0.427894	2.666428	0.070884
H	0.281315	-2.637893	-0.105116
H	2.568771	-2.151391	-0.063213
H	3.617349	-0.077377	0.033660
H	2.684367	2.051795	0.098018
H	-2.149325	2.258424	-0.014905
H	-3.754113	0.139805	-0.118685
H	-2.272624	-2.077799	-0.206290
H	-2.201031	-1.646991	2.022201

Frequencies:

-209.6463	151.2600	164.5548
215.6334	239.4381	321.7061
339.7702	411.9204	427.9588
499.9117	574.4010	609.2386
680.5574	727.7967	744.4871
747.2399	789.9642	815.4755
834.4624	876.7282	914.4922
939.0032	961.5693	975.6800
994.0541	1010.1070	1027.3321
1063.7632	1080.1893	1182.7574
1236.4578	1243.3312	1290.5692
1322.6315	1336.2847	1418.6381
1422.8863	1479.7087	1486.8400
1523.4603	1578.6575	1624.9718
1641.8327	3132.8318	3135.6252
3142.6867	3161.0842	3169.7890
3202.1020	3218.7098	3230.5830

T1 diagnostic:

0.01306784

ts4

Coordinates:

C	-1.290042	1.402102	0.046057
C	1.253427	-1.355938	-0.042243

C	-1.237357	-1.394194	-0.068217
C	-2.437760	-0.723187	-0.046207
C	-2.464036	0.688400	0.014100
C	-0.035710	0.739602	0.018314
C	1.195175	1.449180	0.023361
C	2.397911	0.782533	-0.046104
C	2.430979	-0.623417	-0.102225
C	-0.011778	-0.687789	-0.035574
H	-1.307449	2.486124	0.089980
H	1.272031	-2.428367	-0.198449
H	-1.215993	-2.478359	-0.104629
H	-3.369923	-1.276121	-0.072520
H	-3.416244	1.206388	0.034545
H	1.171756	2.533084	0.065342
H	3.327173	1.340625	-0.064286
H	3.382405	-1.138825	-0.163082
H	1.351379	-1.908297	1.845530

Frequencies:

-650.1622	169.4711	177.4633
274.4128	346.5521	372.1822
413.7967	482.4532	510.9278
517.9477	520.5842	628.9759
638.2764	736.7042	772.2177
787.6741	800.1493	807.7009
862.6898	900.9492	948.1820
961.1559	979.4696	995.5094
1002.7252	1036.8730	1048.2755
1145.3348	1166.2804	1169.2303
1184.7330	1226.9676	1265.3314
1285.2661	1382.1860	1390.3713
1415.7753	1484.4935	1488.4580
1543.7243	1599.6590	1623.1188
1661.4634	3158.5775	3161.3291
3163.8446	3169.6611	3176.2030
3178.9315	3188.7473	3191.1775

T1 diagnostic:

0.01217186

ts5

Coordinates:

C	0.572149	1.603450	0.007093
C	0.556775	-1.590091	-0.067089
C	1.906807	-1.264888	-0.098101
C	2.507319	-0.003514	-0.021023

C	1.924784	1.262947	0.031296
C	-0.538440	0.766198	0.004945
C	-1.878614	1.165444	-0.080013
C	-2.695081	0.021370	-0.091626
C	-1.907307	-1.126582	-0.005384
C	-0.545346	-0.736170	0.080487
H	0.351401	2.668105	-0.019861
H	0.317926	-2.648934	-0.129764
H	2.590866	-2.103275	-0.186440
H	3.593870	-0.010727	-0.027543
H	2.619012	2.097111	0.058406
H	-2.218355	2.190676	-0.127644
H	-3.775057	0.029996	-0.160094
H	-2.257140	-2.149046	-0.000860
H	-0.640797	-0.662892	2.030285

Frequencies:

-755.9722	157.6650	175.0018
284.5091	333.4974	348.9330
402.4831	414.2563	429.8079
502.1292	576.0866	607.1978
670.5024	711.6758	735.6976
748.0401	779.2869	798.6480
830.1590	870.4881	914.1363
941.5654	957.9397	960.5852
987.8323	1006.3436	1037.5846
1064.4377	1084.8984	1181.9894
1239.1812	1242.6692	1289.1415
1313.8044	1332.9508	1402.4545
1423.6533	1481.6550	1490.3607
1515.6323	1569.9798	1617.1070
1631.7838	3132.4742	3135.9923
3143.1289	3159.4845	3168.6342
3198.5646	3215.7394	3225.7835

T1 diagnostic:

0.01394631

ts6

Coordinates:

C	0.020771	-0.679092	-0.090075
C	0.089070	0.725772	0.016176
C	1.213256	-1.420486	-0.149112
C	-1.306768	-1.243653	-0.119318
C	-2.387291	0.454854	-0.187986
C	-1.227866	1.418050	-0.216556

C	2.436541	-0.774399	-0.023664
C	2.494029	0.612336	0.146510
C	1.317937	1.361309	0.156317
C	-2.418452	-0.657721	0.659311
H	1.173307	-2.499540	-0.252273
H	-1.506739	-2.133612	-0.709424
H	-3.261376	0.745922	-0.775452
H	-1.204005	1.945129	-1.177778
H	-1.423768	2.184422	0.546950
H	3.353741	-1.352593	-0.047651
H	3.452948	1.107756	0.246074
H	1.364816	2.442901	0.238376
H	-3.336290	-1.222210	0.781567

Frequencies:

-787.2783	139.2284	173.1309
289.2684	354.2698	414.2939
453.0258	496.9035	537.9145
591.8230	622.1080	683.6507
722.7211	760.5477	780.2137
803.4382	814.5674	861.6147
906.6723	925.1670	950.2875
980.4645	985.0293	1045.6898
1067.3421	1126.0966	1162.7347
1169.5608	1177.0651	1184.8849
1213.9667	1263.5601	1284.4062
1329.4274	1349.4111	1356.1286
1445.4574	1457.9209	1493.0568
1505.3360	1599.0450	1631.4179
3000.2588	3031.0566	3068.6693
3141.1353	3153.2612	3160.4244
3164.5490	3173.7545	3186.6945

T1 diagnostic:

0.01525239

ts7

Coordinates:

C	0.896778	1.637157	0.071082
C	0.183851	-1.405194	-0.563511
C	1.514013	-1.217261	-0.658334
C	2.371810	-0.366937	0.160418
C	2.103736	0.922526	0.472432
C	-0.304930	1.083364	-0.034530
C	-1.645381	1.233086	-0.486008
C	-2.517979	0.230136	-0.195342

C	-2.008871	-0.834852	0.644893
C	-0.686370	-1.189801	0.624560
H	1.032417	2.691054	-0.199431
H	-0.300759	-1.941869	-1.377463
H	2.017193	-1.697856	-1.496239
H	3.365496	-0.745701	0.388686
H	2.887996	1.513108	0.938083
H	-1.959674	2.125379	-1.026277
H	-3.567478	0.298573	-0.459671
H	-2.621530	-1.141615	1.488981
H	-0.293591	-1.654424	1.529375

Frequencies:

-474.4738	131.1082	165.4581
215.8135	225.4796	301.0705
353.0853	403.0544	449.9351
521.2385	543.6334	607.6556
652.7027	733.2331	746.5873
768.0510	799.0064	802.0963
853.5082	909.7348	937.4564
943.3183	962.7823	979.4731
984.7798	1000.3246	1043.2357
1070.9934	1152.9097	1214.6418
1221.9229	1261.7849	1271.5262
1360.3590	1390.5418	1423.1831
1448.1645	1491.3649	1567.5293
1598.6914	1660.9573	1700.9419
3016.6413	3098.4667	3104.5880
3105.8346	3123.5105	3129.4075
3137.8425	3151.0803	3178.2980

T1 diagnostic:

0.01636126

ts8

Coordinates:

C	0.744150	1.538491	-0.304598
C	0.162805	-1.113812	0.645950
C	1.530787	-1.068268	0.769655
C	2.392429	-0.329984	-0.122942
C	2.009621	0.870361	-0.621151
C	-0.253772	1.036767	0.408020
C	-0.646379	-1.327612	-0.603048
C	-1.868225	-0.796866	-0.829441
C	-2.454409	0.232067	0.018241
C	-1.631449	1.093645	0.691159

H	0.630370	2.546689	-0.710673
H	-0.370665	-1.456208	1.534655
H	1.969400	-1.418924	1.701741
H	3.412658	-0.669271	-0.282200
H	2.705430	1.433552	-1.234107
H	-0.263906	-2.084034	-1.281875
H	-2.450800	-1.173941	-1.665065
H	-3.530888	0.278494	0.143396
H	-2.014952	1.734923	1.483055

Frequencies:

-795.1597	135.7097	162.5902
226.8629	264.1601	344.5246
346.0516	392.7500	424.8003
478.4421	532.4686	614.6265
645.0971	702.1444	739.1559
767.4256	796.8421	821.0579
864.1518	874.7205	916.0116
934.5399	948.0528	961.7097
975.8804	991.5450	1028.1711
1065.9515	1150.5649	1200.9597
1213.3422	1243.7870	1296.2985
1360.9113	1385.5828	1418.4923
1448.1456	1460.9321	1530.0695
1586.9844	1607.6646	1734.6941
3058.0344	3082.4779	3112.6406
3122.6328	3137.2156	3139.7282
3159.1754	3169.4602	3171.3959

T1 diagnostic:

0.01703992

ts9

Coordinates:

H	-0.775989	-1.823722	0.000000
C	-0.709435	-0.142945	0.000000
C	0.738875	-0.037416	0.000000
C	1.415255	-0.006117	1.242480
C	1.415255	-0.006117	-1.242480
C	0.720529	0.046825	2.427586
C	0.720529	0.046825	-2.427586
C	-0.698378	0.102799	2.427924
C	-0.698378	0.102799	-2.427924
C	-1.395746	0.037084	1.255222
C	-1.395746	0.037084	-1.255222
H	2.500445	0.000623	1.244188

H	2.500445	0.000623	-1.244188
H	1.255826	0.083486	3.369454
H	1.255826	0.083486	-3.369454
H	-1.226986	0.209897	3.368499
H	-1.226986	0.209897	-3.368499
H	-2.479573	0.075387	1.248852
H	-2.479573	0.075387	-1.248852

Frequencies:

-1051.2326	167.5063	173.6728
349.7147	376.8221	458.7492
478.0643	493.3704	521.1953
556.4473	568.4021	622.8488
638.7180	724.8391	745.9743
783.5204	802.7391	812.5422
838.2554	903.3335	942.9577
956.5109	973.9291	988.8651
994.1490	1027.1974	1036.7522
1149.0682	1158.7712	1176.1958
1181.2693	1217.4054	1264.9950
1277.1688	1378.8420	1383.6449
1412.7369	1478.4869	1485.2595
1532.4412	1587.5627	1637.9995
1656.2151	3157.8904	3160.0168
3163.8321	3166.2547	3177.1255
3178.6405	3188.8735	3190.0081

T1 diagnostic:

0.01344706

ts11

Coordinates:

C	0.843982	1.436882	0.168057
C	0.145555	-1.037999	-0.511199
C	1.557552	-1.264190	-0.514147
C	2.457271	-0.397529	0.041599
C	2.100477	0.952405	0.363314
C	-0.303235	0.589077	-0.076392
C	-1.583915	1.025451	-0.633965
C	-2.602706	0.183663	-0.174524
C	-2.083193	-0.787645	0.667243
C	-0.611310	-0.633451	0.744503
H	0.670717	2.508222	0.168790
H	-0.460477	-1.494429	-1.286510
H	1.920272	-2.132049	-1.057380
H	3.508425	-0.664879	0.058605

H	2.898199	1.643240	0.614215
H	-1.697758	1.856220	-1.317130
H	-3.644076	0.264149	-0.459574
H	-2.636074	-1.578698	1.154938
H	-0.082090	-0.801766	1.677108

Frequencies:

-280.4912	181.4816	218.5521
262.7172	383.7532	441.8147
466.7069	537.2675	565.2450
631.4128	666.4176	688.8338
706.0923	751.4119	774.4233
817.6621	840.8765	895.4859
919.0596	936.8478	945.4581
970.2781	975.7107	995.5383
1020.8965	1054.6139	1093.0591
1099.8055	1106.9097	1178.3897
1203.9027	1208.0273	1244.1848
1287.2609	1340.9176	1359.9620
1397.9555	1432.7566	1468.3069
1491.2422	1528.2216	1639.1199
3139.0966	3142.4671	3153.1384
3157.9321	3166.5916	3179.3568
3192.1694	3209.2862	3220.1824

T1 diagnostic:

0.01316850

ts13

Coordinates:

C	-0.851923	1.407867	0.293337
C	-0.106375	-0.835023	-0.615359
C	-1.537713	-1.215858	-0.533105
C	-2.463615	-0.391812	0.002254
C	-2.125365	0.942985	0.439160
C	0.195253	0.580778	-0.224033
C	0.716752	-0.999210	0.669519
C	2.105059	-0.785358	0.668252
C	2.563430	0.271264	-0.175245
C	1.540272	1.075374	-0.590199
H	-0.604120	2.429913	0.561848
H	0.415649	-1.205729	-1.500399
H	-1.829615	-2.178554	-0.940104
H	-3.499686	-0.709422	0.057091
H	-2.901366	1.583668	0.840266
H	0.191342	-1.400769	1.527800

H	2.743923	-1.269571	1.398056
H	3.608292	0.457842	-0.387996
H	1.660923	1.986584	-1.164050

Frequencies:

-709.3304	157.9102	199.5425
264.0655	382.3071	430.0475
443.8636	512.0984	580.9963
604.8530	652.7695	679.3560
693.7008	713.9057	742.2758
799.1996	820.9862	857.0589
900.8864	933.2384	954.0272
977.9378	992.6941	998.3463
1027.5960	1040.3123	1103.5917
1125.7387	1150.9665	1172.5240
1197.3676	1225.4035	1271.5901
1302.4655	1349.3763	1375.4717
1399.1799	1423.8628	1471.5372
1522.6658	1537.5275	1657.3558
3048.5274	3152.8572	3159.4816
3166.3880	3172.6064	3178.0161
3181.7024	3190.4387	3202.9770

T1 diagnostic:

0.01367120

References

(1) Bogojeski, M.; Vogt-Maranto, L.; Tuckerman, M. E.; Müller, K.-R.; Burke, K. Quantum chemical accuracy from density functional approximations via machine learning. *Nat. Commun.* **2020**, *11*, 5223.

(2) Fujioka, K.; Lam, E.; Loi, B.; Sun, R. Ab initio molecular dynamics benchmarking study of machine-learned potential energy surfaces for the HBr^+ + HCl reaction. *Carbon Trends* **2023**, *11*, 100257.

(3) He, C.; Yang, Z.; Doddipatla, S.; Thomas, A. M.; Kaiser, R. I.; Galimova, G. R.; Mebel, A. M.; Fujioka, K.; Sun, R. Directed gas phase preparation of ethynylallene ($H_2CCCHCCH$; X^1A') via the crossed molecular beam reaction of the methylidyne radical (CH; $X^2\Pi$) with vinylacetylene ($H_2CCHCCH$; X^1A'). *Phys. Chem. Chem. Phys.* **2022**, *24*, 26499-26510.

(4) McQuarrie, D. A. *Problems and Solutions to Accompany McQuarrie and Simon's Physical Chemistry*; University Science Books, 1998.

(5) Hase, W. L. Unimolecular and intramolecular dynamics. Relationship to potential energy surface properties. *J. Phys. Chem.* **1986**, *90*, 365-374.

(6) Bhuiyan, L. B.; Hase, W. L. Sum and density of states for anharmonic polyatomic molecules. Effect of bend–stretch coupling. *J. Chem. Phys.* **1983**, *78*, 5052-5058.

(7) Kaiser, R. I.; Maksyutenko, P.; Ennis, C.; Zhang, F.; Gu, X.; Krishtal, S. P.; Mebel, A. M.; Kostko, O.; Ahmed, M. Untangling the chemical evolution of Titan's atmosphere and surface–from homogeneous to heterogeneous chemistry. *Faraday Discuss.* **2010**, *147*, 429-478.

(8) Yang, Z.; Doddipatla, S.; He, C.; Krasnoukhov, V. S.; Azyazov, V. N.; Mebel, A. M.; Kaiser, R. I. Directed gas phase formation of silene (H_2SiCH_2). *Chem. Eur. J.* **2020**, *26*, 13584-13589.

(9) Kaiser, R. I.; Gu, X.; Zhang, F.; Maksyutenko, P. Crossed beam reactions of methylidyne [$CH(X^2\Pi)$] with D2-acetylene [$C_2D_2(X^1\Sigma_g^+)$] and of D1-methylidyne [$CD(X^2\Pi)$] with acetylene [$C_2H_2(X^1\Sigma_g^+)$]. *Phys. Chem. Chem. Phys.* **2012**, *14*, 575-588.

(10) Maksyutenko, P.; Zhang, F.; Gu, X.; Kaiser, R. I. A crossed molecular beam study on the reaction of methylidyne radicals [$CH(X^2\Pi)$] with acetylene [$C_2H_2(X^1\Sigma_g^+)$]—competing C_3H_2 + H and C_3H + H_2 channels. *Phys. Chem. Chem. Phys.* **2011**, *13*, 240-252.

(11) Daly, N. R. Scintillation type mass spectrometer ion detector. *Rev. Sci. lnstrum.* **1960**, *31*, 264-267.

(12) Brink, G. O. Electron bombardment molecular beam detector. *Rev. Sci. lnstrum.* **1966**, *37*, 857-860.

(13) Levine, R. D. *Molecular Reaction Dynamics*; Cambridge University Press: Cambridge, U.K., 2005.

(14) Becke, A. D. A new mixing of Hartree–Fock and local density‐functional theories. *J. Chem. Phys.* **1993**, *98*, 1372-1377.

(15) Lee, C.; Yang, W.; Parr, R. G. Development of the Colle-Salvetti correlation-energy formula into a functional of the electron density. *Phys. Rev. B* **1988**, *37*, 785.

(16) Curtiss, L. A.; Raghavachari, K.; Redfern, P. C.; Rassolov, V.; Pople, J. A. Gaussian-3 (G3) theory for molecules containing first and second-row atoms. *J. Chem. Phys.* **1998**, *109*, 7764-7776.

(17) Baboul, A. G.; Curtiss, L. A.; Redfern, P. C.; Raghavachari, K. Gaussian-3 theory using density functional geometries and zero-point energies. *J. Chem. Phys.* **1999**, *110*, 7650-7657.

(18) Curtiss, L. A.; Raghavachari, K.; Redfern, P. C.; Baboul, A. G.; Pople, J. A. Gaussian-3 theory using coupled cluster energies. *Chem. Phys. Lett.* **1999**, *314*, 101-107.

(19) Jin, H.; Mebel, A. M.; Farooq, A. Acetylene addition to the fulvenallenyl moiety in aromatic hydrocarbons. *Proc. Combust. Inst.* **2024**, *40*, 105777.

(20) Frisch, M. J.; Trucks, G. W.; Schlegel, H. B.; Scuseria, G. E.; Robb, M. A.; Cheeseman, J. R.; Scalmani, G.; Barone, V.; Mennucci, B.; Petersson, G. A.; et al. *Gaussian 16* Revision C. 1; Gaussian Inc.: Wallingford, CT, 2019.

(21) Werner, H. J.; Knowles, P. J.; Lindh, R.; Manby, F. R.; Schütz, M.; Celani, P.; Korona, T.; Rauhut, G.; Amos, R.; Bernhardsson, A.; et al. *MOLPRO, version 2021.2 A Package of Ab Initio Programs;* University of Cardiff: Cardiff, UK, 2021.

(22) Robinson, P. J.; Holbrook, K. A. *Unimolecular Reactions*; Wiley-Interscience: London and New York, 1972.

(23) Eyring, H.; Lin, S. H.; Lin, S. M. *Basic Chemical Kinetics*; John Wiley & Sons, New York, Chichester, Brisbane, Toronto, 1980.

(24) Steinfeld, J. I.; Francisco, J. S.; Hase, W. L. *Chemical Kinetics and Dynamics*; Prentice Hall: Upper Saddle River, NJ, 1999.

(25) Kislov, V. V.; Nguyen, T. L.; Mebel, A. M.; Lin, S. H.; Smith, S. C. Photodissociation of benzene under collision-free conditions: An ab initio/Rice–Ramsperger–Kassel–Marcus study. *J. Chem. Phys.* **2004**, *120*, 7008-7017.

(26) He, C.; Zhao, L.; Thomas, A. M.; Morozov, A. N.; Mebel, A. M.; Kaiser, R. I. Elucidating the chemical dynamics of the elementary reactions of the 1-propynyl radical (CH_3CC; X^2A_1) with methylacetylene (H_3CCCH; X^1A_1) and allene (H_2CCCH_2; X^1A_1). *J. Phys. Chem. A* **2019**, *123*, 5446-5462.

(27) Mebel, A. M.; Landera, A.; Kaiser, R. I. Formation mechanisms of naphthalene and indene: From the interstellar medium to combustion flames. *J. Phys. Chem. A* **2017**, *121*, 901-926.

(28) Mebel, A. M.; Li, W.; Pratali Maffei, L.; Cavallotti, C.; Morozov, A. N.; Wang, C.-Y.; Yang, J.-Z.; Zhao, L.; Kaiser, R. I. Fulvenallenyl radical ($C_7H_5^{\cdot}$)-mediated gas-phase synthesis of bicyclic aromatic $C_{10}H_8$ isomers: can fulvenallenyl efficiently react with closed-shell hydrocarbons? *J. Phys. Chem. A* **2024**, *128*, 5707-5720.

(29) Lourderaj, U.; Sun, R.; Kohale, S. C.; Barnes, G. L.; De Jong, W. A.; Windus, T. L.; Hase, W. L. The VENUS/NWChem software package. Tight coupling between chemical dynamics simulations and electronic structure theory. *Comput. Phys. Commun.* **2014**, *185*, 1074-1080.

(30) Hu, X.; Hase, W. L.; Pirraglia, T. Vectorization of the general Monte Carlo classical trajectory program VENUS. *J. Comput. Chem.* **1991**, *12*, 1014-1024.

(31) Kendall, R. A.; Aprà, E.; Bernholdt, D. E.; Bylaska, E. J.; Dupuis, M.; Fann, G. I.; Harrison, R. J.; Ju, J.; Nichols, J. A.; Nieplocha, J. High performance computational chemistry: An overview of NWChem a distributed parallel application. *Comput. Phys. Commun.* **2000**, *128*, 260-283.

(32) He, C.; Fujioka, K.; Nikolayev, A. A.; Zhao, L.; Doddipatla, S.; Azyazov, V. N.; Mebel, A. M.; Sun, R.; Kaiser, R. I. A chemical dynamics study of the reaction of the methylidyne radical (CH, $X^2\Pi$) with dimethylacetylene (CH_3CCCH_3, X^1A_{1g}). *Phys. Chem. Chem. Phys.* **2022**, *24*, 578-593.

(33) Stephens, P. J.; Devlin, F. J.; Chabalowski, C. F.; Frisch, M. J. Ab initio calculation of vibrational absorption and circular dichroism spectra using density functional force fields. *J. Phys. Chem.* **1994**, *98*, 11623-11627.

(34) Raghavachari, K.; Trucks, G. W.; Pople, J. A.; Head-Gordon, M. A fifth-order perturbation comparison of electron correlation theories. *Chem. Phys. Lett.* **1989**, *157*, 479-483.

(35) Hehre, W. J.; Ditchfield, R.; Pople, J. A. Self—consistent molecular orbital methods. XII. Further extensions of Gaussian—type basis sets for use in molecular orbital studies of organic molecules. *J. Chem. Phys.* **1972**, *56*, 2257-2261.

(36) Fujioka, K.; Weitzel, K.-M.; Sun, R. The potential energy profile of the HBr^+ + HCl bimolecular collision. *J. Phys. Chem. A* **2022**, *126*, 1465-1474.

(37) Fujioka, K.; Kaiser, R. I.; Sun, R. Unsupervised reaction pathways search for the oxidation of hypergolic ionic liquids: 1-ethyl-3-methylimidazolium cyanoborohydride ($EMIM^+/CBH^-$) as a case study. *J. Phys. Chem. A* **2022**, *127*, 913-923.

(38) Zhang, Y.; Xia, J.; Jiang, B. REANN: A PyTorch-based end-to-end multi-functional deep neural network package for molecular, reactive, and periodic systems. *J. Chem. Phys.* **2022**, *156*.

(39) Larsen, A. H.; Mortensen, J. J.; Blomqvist, J.; Castelli, I. E.; Christensen, R.; Dułak, M.; Friis, J.; Groves, M. N.; Hammer, B.; Hargus, C. The atomic simulation environment—a Python library for working with atoms. *J. Phys.: Condens. Matter* **2017**, *29*, 273002.

(40) Heller, S. R.; McNaught, A.; Pletnev, I.; Stein, S.; Tchekhovskoi, D. InChI, the IUPAC international chemical identifier. *J. Cheminform.* **2015**, *7*, 1-34.
